# The Invasive Margin of Glioblastoma as a Molecular Ecosystem: Spatial Heterogeneity, Tumor–Host Interactions, and Therapeutic Opportunities

**DOI:** 10.3390/ijms27167449

**Published:** 2026-08-20

**Authors:** Nikodem Kuczyński, Dawid Larysz, Dorota Uchman-Rzeżnik, Gunawan Irianto, Dawid Pilewski

**Affiliations:** 1 Faculty of Medicine, Collegium Medicum, The Mazovian University in Płock, 09-402 Płock, Poland; n.kuczynski@mazowiecka.edu.pl (N.K.); d.uchman-rzeznik@mazowiecka.edu.pl (D.U.-R.); 2 Department of Head and Neck Surgery for Children and Adolescents, University of Warmia and Mazury in Olsztyn, 10-561 Olsztyn, Poland; dawid.larysz@uwm.edu.pl; 3 Faculty of Health Science, University of Muhammadiyah Pringsewu, Lampung 35373, Indonesia; gunawanirianto@umpri.ac.id; 4 Faculty of Health Science, Collegium Medicum, The Mazovian University in Płock, 09-402 Płock, Poland

**Keywords:** glioblastoma, invasive margin, peritumoral brain zone, spatial heterogeneity, tumor microenvironment, cell-state plasticity, residual disease, treatment resistance

## Abstract

Glioblastoma (GBM) recurs predominantly from infiltrative disease that persists beyond the contrast-enhancing tumor (CET). This structured narrative review aims to define the invasive margin and peritumoral brain zone (PBZ) as a spatially organized molecular ecosystem, summarize the approaches used to interrogate this compartment, and evaluate how malignant-cell plasticity, host niches, and treatment-induced remodeling contribute to minimal residual disease and recurrence. A structured literature search of PubMed/MEDLINE, Scopus, and Web of Science identified the clinical, translational, preclinical, and review literature available through July 2026; evidence was synthesized qualitatively, with priority given to human tissue studies and single-cell or spatially resolved analyses. Across studies, the margin differs from both tumor core and normal brain and contains heterogeneous malignant states interacting with neural, vascular, immune, hypoxic, and extracellular-matrix-supported niches. Surgery, radiotherapy, and systemic treatment further reshape these interactions through inflammation, vascular injury, senescence, hypoxia, and fibrosis. The main translational challenge is therefore not simply to control the CET, but to identify biologically high-risk non-enhancing tissue and demonstrate that therapy reaches and modifies it. We propose three priorities: image-registered characterization of residual compartments, regional measurement of drug exposure and target engagement, and integration of local margin control with distributed and niche-directed treatment. Prospective validation is required before spatial PBZ biomarkers can guide routine care.

## 1. Introduction

Glioblastoma (GBM) is the most common malignant primary brain tumor in adults and remains one of the most aggressive neoplasms of the central nervous system (CNS). Under the 2021 World Health Organization classification, GBM is an isocitrate dehydrogenase (IDH)-wild-type diffuse astrocytic glioma of CNS WHO grade 4, defined by characteristic histopathological or molecular features [1]. Population-based data indicate that GBM accounts for more than half of malignant primary brain and other CNS tumors in adults [2]. Despite advances in classification, imaging, surgery, and supportive care, long-term survival remains uncommon [2,3].

Standard treatment combines maximal safe resection with radiotherapy and temozolomide; tumor-treating fields and other approaches are used in selected settings [4]. Its main limitation is biological rather than purely technical: resection targets the contrast-enhancing tumor (CET), whereas malignant cells can extend into non-enhancing brain along white-matter tracts, perivascular spaces, and relatively preserved neural structures. Thus, the radiological border does not represent the true tumor boundary, and removal of all enhancing tissue does not eliminate the full malignant-cell population [3,4,5].

The peritumoral brain zone (PBZ), including tissue around the resection cavity, is clinically important because recurrence usually arises within or near the treated region. It contains not only sparse infiltrating GBM cells but also tumor-conditioned neural, glial, immune, vascular, stromal, and extracellular-matrix (ECM) elements [6,7]. Their interactions can support survival, migration, immune evasion, stem-like properties, and treatment resistance even when conventional imaging shows no obvious tumor.

Proteomic, single-cell, and spatial multi-omic studies further show that the invasive margin is molecularly distinct from the tumor core and internally heterogeneous [5,8,9]. Local vascular conditions, immune composition, neural architecture, ECM organization, and metabolic stress generate spatially different cellular neighborhoods. Experimental work also shows that resident microglia can reorganize ahead of the invasive front and facilitate infiltrative growth [10]. Together, these findings support invasion as a coordinated adaptation of malignant and host cells rather than passive migration through normal brain.

This ecological view has direct therapeutic implications. Control of the CET leaves residual malignant cells and supportive niches within the PBZ, while surgery, radiotherapy, and systemic treatment can further remodel this compartment through inflammation, vascular injury, senescence, hypoxia, and ECM changes [6,7]. The postoperative PBZ is therefore both a site of residual disease and a treatment-responsive target. Locoregional delivery may improve exposure near the cavity, but durable control also requires strategies that address infiltrating disease beyond the immediate margin [11].

In this review, we examine the GBM invasive margin as a molecular ecosystem. We first define the clinical problem of marginal recurrence and the methods used to study the PBZ. We then synthesize evidence on tumor–host interactions, invasive malignant-cell states, spatial niches, treatment-induced remodeling, and eco-evolutionary recurrence. Finally, we consider imaging biomarkers, translational barriers, and spatially informed therapeutic and clinical-trial strategies.

**Figure 1:**
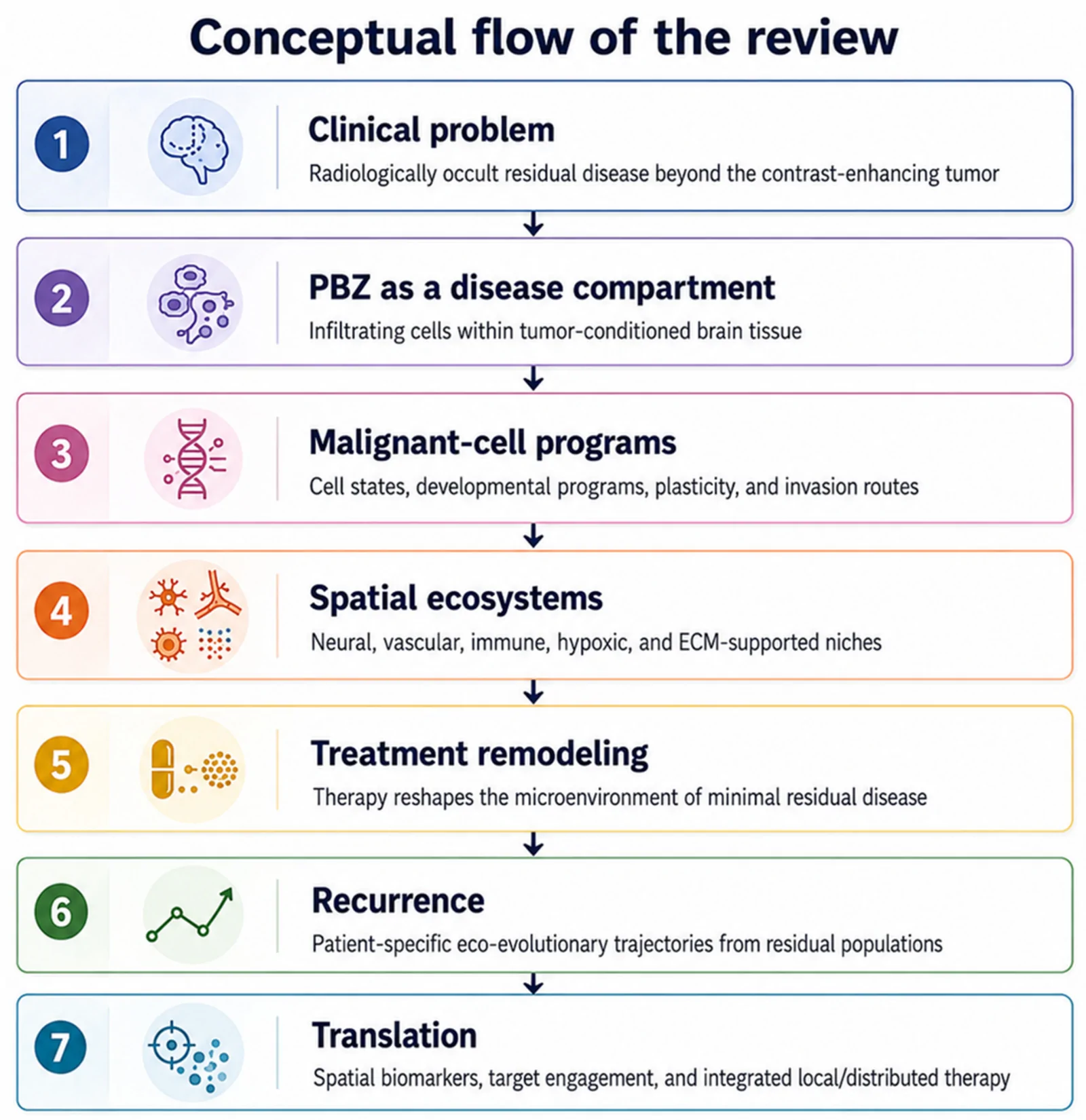
Conceptual flow of the review. Abbreviations: ECM, extracellular matrix; PBZ, peritumoral brain zone.

The organization of the review, from the clinical problem of residual infiltrative disease to spatial mechanisms and translational strategies, is summarized in Figure 1.

## 2. Methods

This study was conducted as a structured narrative review of the literature on the invasive margin of GBM, with particular emphasis on the PBZ, spatial heterogeneity, tumor–host interactions, treatment resistance, and emerging therapeutic strategies. A structured search of PubMed/MEDLINE, Scopus, and Web of Science was performed to identify relevant publications available through July 2026. Search terms included combinations of “glioblastoma,” “invasive margin,” “peritumoral brain zone,” “leading edge,” “tumor microenvironment,” “spatial heterogeneity,” “single-cell sequencing,” “spatial transcriptomics,” “treatment resistance,” and “recurrence.”

Eligible sources included clinical, observational, translational, and preclinical studies, as well as systematic reviews, meta-analyses, and selected narrative reviews relevant to the scope of the article. Human GBM studies were prioritized, while studies involving experimental models or other diffuse gliomas were included when they provided relevant mechanistic evidence. Conference abstracts without available full texts and publications lacking direct relevance to the topic were excluded.

Titles and abstracts were screened, followed by full-text assessment. Priority was given to recent, peer-reviewed studies, human tissue analyses, larger cohorts, and single-cell or spatially resolved investigations. Reference lists of included publications were also searched manually. Two authors independently performed study selection, and disagreements were resolved by consensus. The findings were synthesized qualitatively. Due to the narrative nature of this review, no formal meta-analysis or quantitative synthesis was performed.

Because this was a narrative rather than a systematic review, no PRISMA flow diagram, formal risk-of-bias assessment, or certainty-of-evidence grading was undertaken.

### Approaches Used to Study the PBZ and Post-Treatment Residual Disease

Human studies of the invasive margin rely on spatially annotated tissue rather than on bulk tumor alone. Image-registered multiregion sampling uses preoperative imaging and intraoperative neuronavigation to record the coordinates of separate specimens from the CET, nCET/PBZ, leading edge, and, when clinically indicated, recurrent disease. Histopathological, genomic, transcriptomic, proteomic, and pharmacokinetic measurements can then be mapped back to the anatomical compartment, reducing bias from pooling spatially distinct tissue [12,13,14,15].

Dissociation-based single-cell and single-nucleus RNA sequencing resolves malignant and non-malignant cell states but loses tissue architecture. Spatial transcriptomics, spatial proteomics, imaging mass cytometry, multiplexed imaging, and integrated multi-omics preserve or reconstruct cellular neighborhoods and are therefore particularly useful for studying ecological organization and treatment-associated remodeling [16,17,18,19,20]. Paired primary–recurrent samples add temporal information, although repeat tissue is available mainly from patients undergoing clinically indicated re-resection or biopsy rather than routine serial sampling [21,22,23,24].

Functional studies and phase 0 or window-of-opportunity trials complement descriptive profiling. Short preoperative drug exposure followed by image-localized sampling can measure regional drug concentration and target engagement [15,25]. Between operations, MRI and selected metabolic or PET approaches provide non-invasive longitudinal context, but they cannot fully replace tissue for genomic or single-cell analysis [26,27,28].

## 3. The Clinical Challenge of Marginal Recurrence in Glioblastoma

### 3.1. Standard Therapy and Residual Infiltrative Disease

Glioblastoma treatment is still centered on maximal safe resection followed by radiotherapy and temozolomide [29,30]. Although the extent of resection is clinically important and is associated with improved outcome, surgery is mainly directed against the CET mass. This creates a major limitation, because GBM is a diffusely infiltrative disease that can extend beyond the radiologically visible lesion. Even when postoperative magnetic resonance imaging (MRI) confirms gross total resection of the enhancing component, residual tumor cells may remain in the surrounding brain tissue, where they can later initiate recurrence [31,32,33]. Fluorescence-guided surgery with 5-aminolevulinic acid (5-ALA) has improved intraoperative visualization of malignant glioma tissue and may facilitate a greater extent of resection. However, its value is mainly related to the metabolically active tumor bulk, whereas infiltrating tumor cells in the PBZ may show absent or low fluorescence [34]. 5-ALA improves the surgical control of visible tumor tissue but does not fully overcome the biological problem of non-fluorescent infiltrative disease at the margin.

Complete macroscopic resection is further limited by the involvement of eloquent brain areas and by the risk of neurological impairment, making removal of the entire infiltrative field impossible in many patients [35,36].

### 3.2. Recurrence at the Peritumoral Margin

The clinical relevance of the PBZ is supported by recurrence patterns. Most recurrences are local; reported proportions vary with definitions, imaging, treatment, and follow-up, but many series identify the dominant failure pattern within approximately 1–2 cm of the resection cavity or within the surrounding peritumoral region, even after gross total resection and adjuvant chemoradiotherapy [37,38,39]. These observations suggest that the postoperative margin is not merely an anatomical boundary left after surgery, but a clinically critical compartment in which residual disease may persist and eventually regrow [40,41].

In this context, the postoperative peritumoral compartment comprises tissue that remains after removal of the enhancing tumor bulk and is therefore a principal biological target of postoperative treatment. This shifts the clinical question from whether the visible tumor has been removed to whether the residual peritumoral compartment has been adequately controlled.

### 3.3. Radiologically Occult but Biologically Altered Tissue

A major clinical challenge is that the PBZ may appear non-enhancing on T1-weighted gadolinium-enhanced MRI and macroscopically similar to normal brain tissue. However, the absence of contrast enhancement does not exclude tumor-cell infiltration or tumor-associated molecular alterations [42]. The PBZ has been described as a non-enhancing area surrounding the tumor that may contain specific radiological, cellular, molecular, and metabolic abnormalities [43,44,45]. These changes are not limited to the immediate border of the contrast-enhancing lesion and may support tumor-cell proliferation, invasion and recurrence.

The limitations of conventional imaging are particularly relevant in the fluid-attenuated inversion recovery (FLAIR)/T2 abnormal peritumoral region. FLAIR alterations may extend well beyond the CET, but their borders are often poorly defined and may reflect a mixture of edema, gliosis, vascular changes, inflammatory responses and infiltrating tumor cells. Moreover, complete resection of FLAIR-abnormal tissue is frequently not feasible when this region overlaps with eloquent cortex or critical white-matter pathways. Therefore, the non-enhancing margin represents a clinically ambiguous zone: it may not fulfill radiological criteria for residual enhancing tumor, yet it may still contain biologically relevant residual disease [46,47].

The PBZ should not be regarded simply as edematous or normal adjacent brain. This region may contain infiltrating tumor cells, angiogenesis-related endothelial cells, reactive astrocytes, glioma-associated microglia/macrophages, exhausted tumor-infiltrating lymphocytes and glioma-associated stromal cells [36,48]. Overt tumor cells are detected only in a subset of PBZ samples, but biological abnormalities may also be present in apparently non-infiltrated tissue [36]. At the genomic and transcriptomic levels, the PBZ shows an intermediate profile between tumor core and healthy brain, with alterations involving angiogenesis, extracellular matrix organization, cellular senescence, stemness-related features and tumor suppressor pathways [49,50].

**Figure 2:**
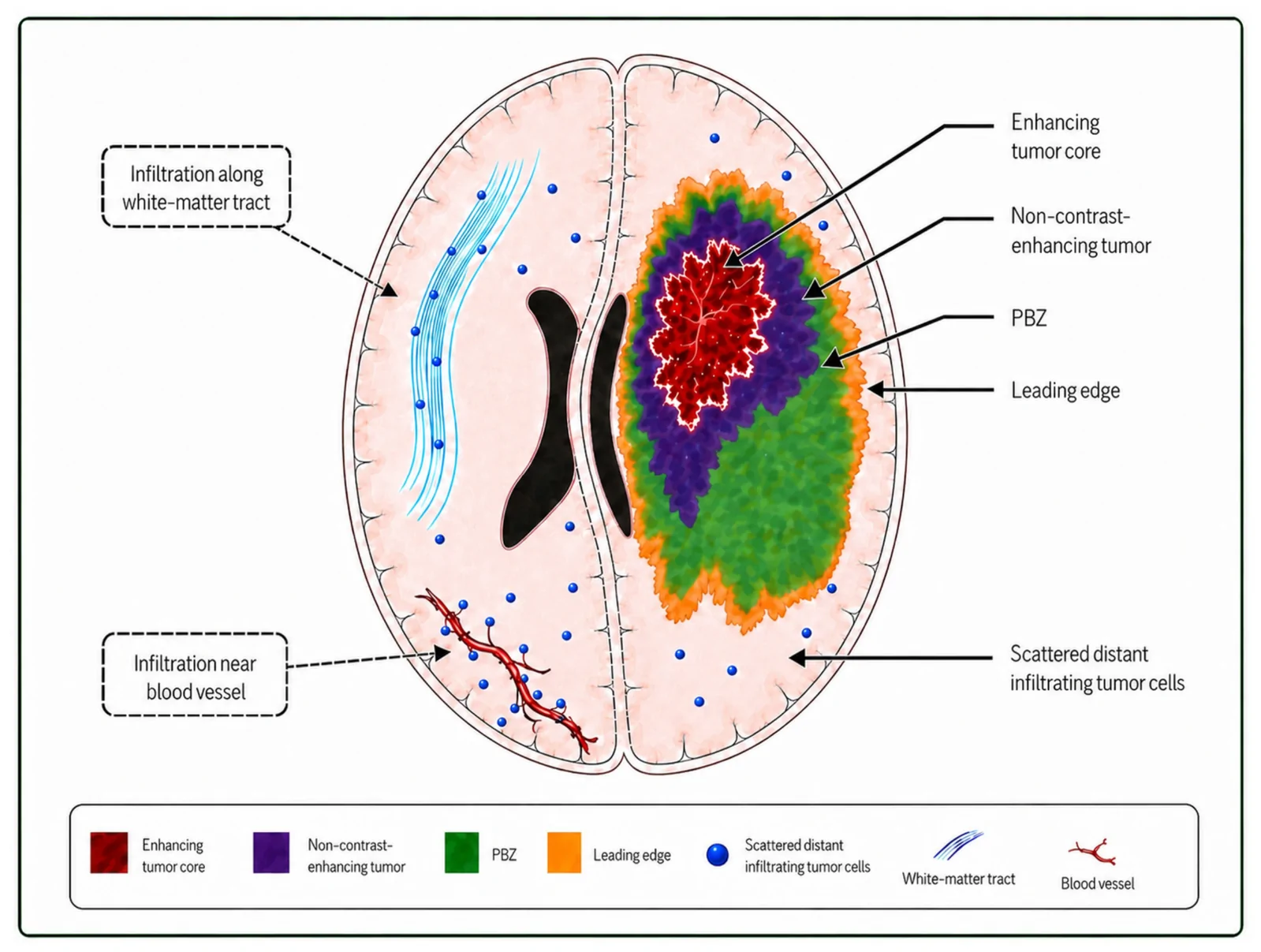
Spatial organization of glioblastoma infiltration beyond the radiologically visible tumor. The schematic illustrates the CET core, nCET, PBZ, and leading edge. Scattered infiltrating tumor cells may extend into the surrounding brain beyond these regions, including along white-matter tracts and in proximity to blood vessels. The illustrated compartments have irregular, partially overlapping boundaries and are not drawn to scale. Abbreviations: CET, contrast-enhancing tumor; nCET, non-contrast-enhancing tumor; PBZ, peritumoral brain zone.

Because the radiological boundaries of glioblastoma do not fully reflect its biological extent, the major spatial compartments associated with tumor infiltration and residual disease are illustrated in Figure 2.

### 3.4. Spatial Heterogeneity and Candidate Imaging Biomarkers Within the PBZ

The PBZ is not a uniform compartment. Recent multiregional analyses have shown that peritumoral heterogeneity extends beyond the tumor core into the surrounding brain parenchyma [51,52,53]. In particular, regions with relatively higher cerebral blood flow have been identified within the PBZ. These “higher cerebral blood flow interfaces” show more neovascularization than lower-flow PBZ regions, increased macrophage and T-cell infiltration, and a higher burden of tumor cells with distinct gene-expression profiles [29]. This suggests that recurrence risk may not be evenly distributed throughout the peritumoral zone. Instead, specific tumor–host interface regions may provide vascular, immune and molecular conditions that favor residual tumor-cell persistence and postoperative regrowth.

Several approaches are being evaluated as candidate biomarkers of biologically high-risk non-enhancing tissue. Perfusion MRI, diffusion-based maps, metabolic imaging, multiparametric nCET segmentation, and amino-acid PET can identify regional abnormalities beyond contrast enhancement [13,26,27,29,54]. Image-registered studies indicate that some of these regions have vascular or molecular features distinct from adjacent brain, but no modality yet provides a universally validated boundary between infiltrating tumor and edema, gliosis, or treatment effect [13,26,27,29,55]. Their current value is therefore strongest for defining high-risk regions for targeted sampling, treatment planning, and prospective validation rather than treating the entire FLAIR abnormality as biologically equivalent disease.

### 3.5. The Margin as a Biological Disease Compartment

Marginal recurrence should not be viewed only as a technical consequence of incomplete tumor removal. Rather, it reflects the biology of GBM as an infiltrative and spatially organized disease, in which residual tumor cells and tumor-conditioned host tissue may persist beyond the contrast-enhancing lesion. Two non-mutually exclusive mechanisms may contribute to this process: sparse infiltrating tumor cells may remain undetected within the surrounding brain parenchyma, while tumor–host crosstalk may reprogram non-neoplastic cells into a permissive microenvironment that supports recurrence even in areas without overt tumor-cell infiltration [29,36]. The invasive margin should be considered an active disease compartment and a critical target for future therapeutic strategies.

## 4. The Glioblastoma Invasive Margin as an Active Tumor–Host Interface

### 4.1. The PBZ as a Transitional Biological Compartment

Microscopically detectable tumor cells are found in only a subset of PBZ samples, estimated at approximately one third of cases [46,47,49].

At the mechanistic level, the defining feature of the PBZ is reciprocal crosstalk between sparse infiltrating GBM cells and non-neoplastic host tissue. Tumor-derived signals can reprogram astrocytic, myeloid, vascular, and stromal populations even where overt neoplastic infiltration is limited [12,49,56,57]. The biological state of the margin therefore depends on both tumor-cell presence and tumor-conditioned host responses.

### 4.2. Cellular Crosstalk and Immune Remodeling at the Tumor–Brain Interface

Infiltrating GBM cells do not migrate through an inert neural background. Instead, they engage in bidirectional communication with astrocytes, microglia, macrophages, vascular cells, and stromal-like populations [58,59,60]. Reactive astrocytes are particularly abundant in areas of tumor invasion and may support GBM-cell survival, proliferation, migration, and resistance to treatment [61,62,63]. Gap-junction communication involving connexin 43 (Cx43) has been implicated in the transfer of protective signals between astrocytes and tumor cells [64,65,66,67], whereas astrocyte-derived tenascin-C and interleukin (IL)-10 may contribute to immune protection [68,69]. Additional soluble mediators have also been implicated in promoting tumor-cell proliferation and invasion. These include IL-6, tumor necrosis factor alpha (TNF-α), transforming growth factor beta (TGF-β), insulin-like growth factor 1 (IGF-1), vascular endothelial growth factor (VEGF), and glial cell line-derived neurotrophic factor [70,71,72,73]. Perivascular astrocytes may also support stem-like phenotypes through osteopontin–cluster of differentiation 44 (CD44) signaling, linking astrocytic remodeling to the maintenance of aggressive GBM-cell states [74,75]. The available evidence supports a role for reactive astrocytes as active regulators of the invasive niche, promoting GBM-cell survival, immune evasion, and the maintenance of stem-like phenotypes.

Microglia and macrophages represent another major component of the tumor–brain interface [76]. Although these cells have traditionally been classified using an M1/M2 polarization model, their functional states in GBM are substantially more complex and should be considered as a continuum rather than a strict dichotomy [77,78,79,80]. Nevertheless, anti-inflammatory and immunosuppressive myeloid programs are frequently observed in the PBZ [81]. Glioma-associated microglia/macrophages may promote extracellular matrix (ECM) degradation, invasion, angiogenesis, and immune escape through the secretion of matrix metalloproteinase-9 (MMP-9), VEGF, TGF-β, IL-10, and other mediators [57,82,83,84]. Crosstalk between myeloid cells and GBM cells may additionally enhance programmed death-ligand 1 (PD-L1) expression in GBM cells, while indoleamine 2,3-dioxygenase (IDO), IL-10, and TGF-β can favor regulatory T-cell activity and impaired cytotoxic T-cell function [85,86,87,88]. The degree of immunosuppression appears to follow a spatial gradient, generally increasing from the peripheral brain toward the tumor core, indicating that the immune landscape of the margin is altered but not identical to that of the central tumor mass [89,90,91].

Glioma-associated stromal cells may further contribute to the formation of a permissive invasive niche [92,93]. Although genetically distinct from neoplastic GBM cells, these stromal-like populations can be recruited and functionally altered by the tumor [94,95]. They have been associated with angiogenic activity, extracellular matrix remodeling, and the induction of phenotypic changes that facilitate tumor-cell migration [96,97,98]. Their interactions with glioma-associated macrophages provide an additional example of how malignant and non-malignant cellular populations cooperate within the PBZ [99].

The principal malignant and non-malignant cellular populations that shape the invasive margin, together with their major tumor-supportive functions and representative mediators, are summarized in Table 1.

### 4.3. Structural Routes and Interface-Level Support

The vasculature of the invasive margin has both trophic and structural functions. In addition to supplying oxygen and nutrients, pre-existing blood vessels provide anatomical routes along which GBM cells can migrate into the surrounding brain [109]. This process, termed vessel co-option, is particularly relevant in the PBZ, where tumor cells may form perivascular cuffs around otherwise normal vessels [102,110,111]. Endothelial-derived signals may attract invasive and stem-like tumor cells toward the perivascular space, which may support their survival and protect them against therapeutic stress [112,113,114]. White-matter tracts provide an additional structural route of dissemination, functioning as longitudinal scaffolds that allow tumor cells to spread over considerable distances, including toward the contralateral hemisphere through the corpus callosum [109].

At the interface, these structural routes are coupled to survival signals. Vessels provide migration scaffolds and angiocrine support, while ECM remodeling can create permissive paths and activate stemness- and resistance-associated programs [74,75,115]. The detailed spatial organization of vascular, hypoxic, immune, neural, and ECM-supported niches is examined in Section 6.

**Figure 3:**
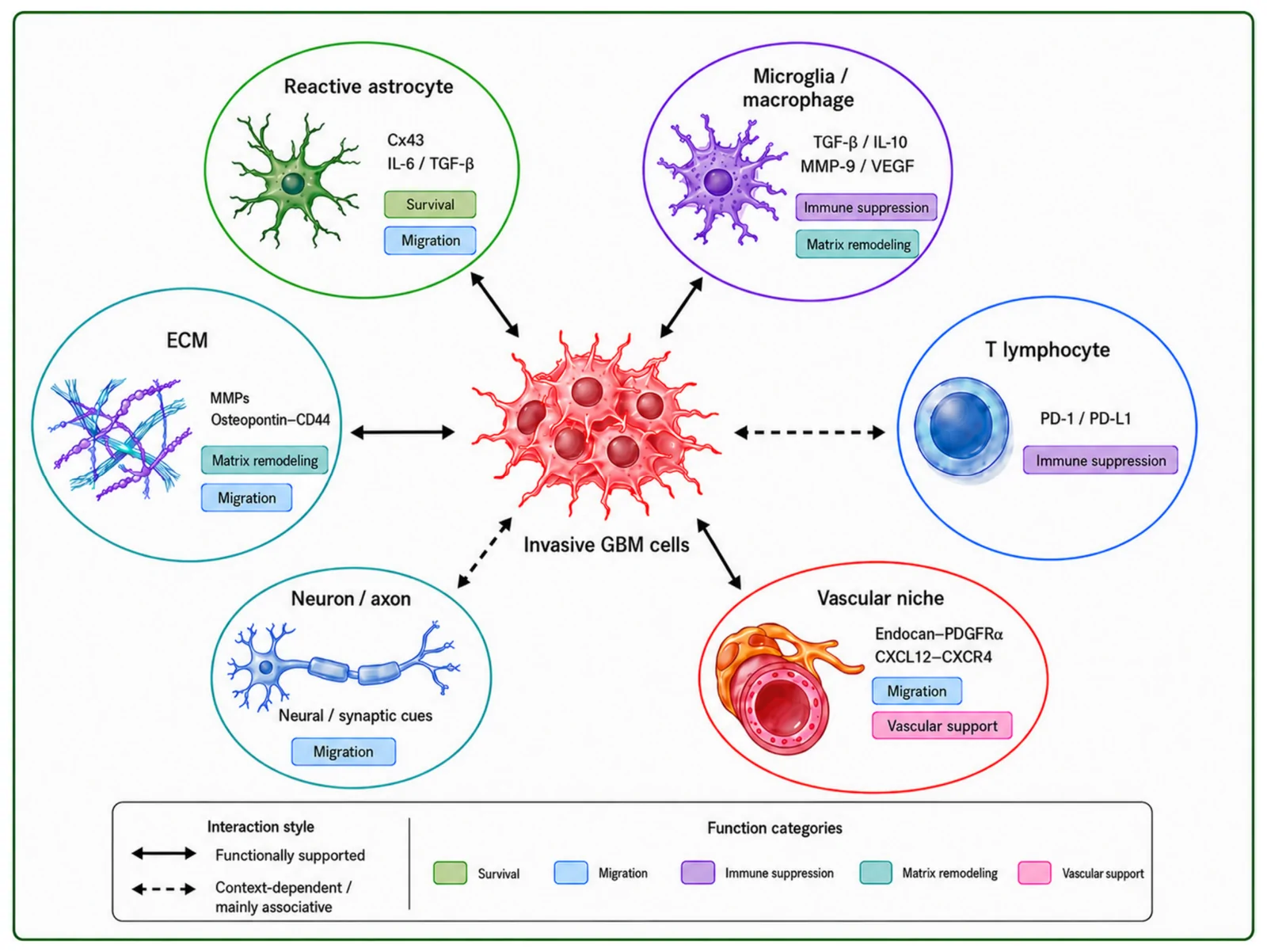
Tumor–host interactions at the glioblastoma invasive margin. The schematic highlights selected interactions between invasive GBM cells and astrocytes, microglia/macrophages, T lymphocytes, the vascular niche, neurons, and the ECM. These interactions support migration, survival, immunosuppression, vascular adaptation, and matrix remodeling. Solid arrows indicate functionally supported interactions, whereas dashed arrows indicate context-dependent or mainly associative relationships. Abbreviations: Cx43, connexin 43; CXCL12, C-X-C motif chemokine ligand 12; CXCR4, C-X-C motif chemokine receptor 4; ECM, extracellular matrix; IL, interleukin; MMP-9, matrix metalloproteinase-9; PD-1, programmed cell death protein 1; PD-L1, programmed death-ligand 1; PDGFRα, platelet-derived growth factor receptor alpha; TGF-β, transforming growth factor beta; VEGF, vascular endothelial growth factor.

The major cellular, vascular, neural, immune, and ECM interactions that shape this tumor–host ecosystem are schematically summarized in Figure 3.

## 5. Infiltrating Glioblastoma Cells Adopt Distinct and Plastic Molecular States

### 5.1. Single-Cell Profiling Reveals Distinct and Heterogeneous Invasive Cell States

The infiltrative component of glioblastoma is not merely a low-density extension of the tumor core. Single-cell RNA sequencing (scRNA-seq) studies indicate that malignant cells at the invasive front display transcriptional programs distinct from those observed in the tumor core. In a single-cell study analyzing cells from the tumor core and adjacent peritumoral brain, infiltrating neoplastic cells shared a recurrent transcriptional signature across patients despite substantial intertumoral heterogeneity [16]. The same analysis also revealed spatial differences in non-malignant populations, including transcriptionally distinct myeloid cells in the tumor core and surrounding peritumoral tissue [16,116]. This evidence suggests that invasion is accompanied by coordinated changes in both malignant cells and their local microenvironment rather than by the passive dispersal of randomly selected tumor cells [16,117].

The molecular identity of infiltrating cells is nevertheless not represented by a single invariant invasive phenotype. Instead, GBM cells occupy a continuum of developmental-like states whose relative abundance differs between tumors and anatomical regions [17,100,118]. The four-state framework proposed by Neftel et al. [100] comprises neural progenitor cell-like (NPC-like), oligodendrocyte progenitor cell-like (OPC-like), astrocyte-like (AC-like), and mesenchymal-like (MES-like) states. Their prevalence is associated with recurrent genetic alterations: cyclin-dependent kinase 4 (*CDK4*) amplification favors NPC-like programs, platelet-derived growth factor receptor alpha (*PDGFRA*) amplification favors OPC-like programs, epidermal growth factor receptor (*EGFR*) alterations are associated with AC-like programs, and neurofibromin 1 (*NF1*) loss promotes MES-like states [100]. Multiple states may coexist within the same tumor, and individual malignant cells can transition between them, demonstrating that transcriptional identity is not rigidly determined by clonal genotype [100,119].

A large integrative single-cell study published in 2025 further refined this model by identifying additional malignant programs, including glial progenitor-like, neuronal-like, and cilia-associated states, and organized GBM heterogeneity into several interconnected layers comprising cell composition, cell-state diversity, pathway activity, and broader tumor-level expression programs [120]. The canonical four-state model remains a useful framework but does not capture the full spectrum of malignant phenotypes present within GBM ecosystems.

The major malignant cell states implicated in glioblastoma invasion, their characteristic molecular features, and their preferential spatial associations are summarized in Table 2.

### 5.2. Stem-like and Developmental Programs Support Invasion

A subset of invasive GBM cells can reactivate developmental programs normally involved in neural progenitor migration and tissue organization [126,127]. Single-cell mapping of primary GBM identified multiple glioblastoma stem-like cell populations within individual tumors, including a population resembling fetal outer radial glia [123,127]. These outer radial glia-like cancer cells exhibited invasive properties and a characteristic mitotic somal translocation behavior associated with radial glial cells during cortical development [127]. These findings suggest that GBM invasion can involve the pathological reactivation of developmental cell-behavior programs rather than reflecting only generic tumor-cell motility [127,128].

Spatial transcriptomic analyses have subsequently demonstrated that stem-like states are distributed non-randomly across glioma tissue [18,129]. Radial glial-like cells were enriched in neuron-rich invasive niches, whereas OPC-like malignant cells were more abundant within the tumor core [129]. Such spatial correlations link cell state directly with local tissue architecture, suggesting that neural, vascular, and immune components may differentially support particular malignant programs in different anatomical niches [124,129]. Accordingly, the phenotype of an invading cell is shaped not only by its genetic background but also by the microenvironment encountered during migration [18,125,129].

Stemness and invasion can therefore be functionally coupled. Stem-like GBM cells combine self-renewal, migration, and stress adaptability, properties that may support survival outside the tumor core and regeneration after treatment [100,127,130,131]. The invasive margin may consequently serve as a reservoir of plastic stem-like cells, although its direct contribution to post-treatment recurrence remains to be demonstrated [18,127,129].

### 5.3. The “Go-or-Grow” Model Should Be Viewed as a Continuum

The relationship between tumor-cell invasion and proliferation has frequently been described using the “go-or-grow” model, according to which glioma cells alternate between a migratory state and a proliferative state [132,133]. Although this concept provides a useful explanation for why sparsely distributed cells at the invasive front may differ from the rapidly expanding tumor core, it should not be interpreted as an absolute biological dichotomy [132,134]. Direct experimental evidence has shown that individual GBM cells may simultaneously possess substantial invasive and proliferative capabilities. These capacities can be coupled within the same cells over the course of tumor progression rather than representing mutually exclusive phenotypes [134].

Recent studies have linked specific transcriptional states to distinct anatomical routes of glioblastoma invasion [107,121,135]. Single-cell profiling and spatial protein analyses of patient-derived xenografts showed that perivascular invasion was associated predominantly with MES-like and OPC-like states, with annexin A1 (ANXA1) identified as a functional regulator of tumor-cell association with vascular structures. By contrast, diffusely invading tumors were enriched for NPC-like and AC-like states and showed increased expression of two candidate regulators, regulatory factor X4 (RFX4) and HOP homeobox (HOPX). Genetic ablation of *ANXA1*, *RFX4*, or *HOPX* altered invasion patterns, redistributed malignant cell states, and prolonged survival in xenografted mice, providing functional evidence that route-specific invasive phenotypes are plastic and experimentally reprogrammable [121].

A 2026 multimodal analysis integrating scRNA-seq, spatial transcriptomics, and multiplexed imaging further distinguished local from distal glioblastoma invasion programs [122,136]. Patient-derived models capable of distal invasion, including spread into the contralateral hemisphere, were enriched in OPC-like cells and predominantly infiltrated along white-matter tracts through peri-axonal routes [107,108,122]. In contrast, models lacking substantial distal spread but exhibiting local invasion had MES-like-enriched tumor cores and preferentially invaded along perivascular routes [121,122]. The baseline transcriptional states associated with a model’s preferred invasion route differed from the distally invading glioma and locally invading glioma transcriptional programs expressed during active invasion [122,136]. A model’s baseline propensity for invasion and the molecular response accompanying active invasion are related but biologically distinct properties [122].

Current evidence indicates that distinct anatomical routes of invasion are associated with partially different malignant cell states and molecular regulators, as summarized in Table 3.

### 5.4. Cell-State Plasticity as a Mechanism of Treatment Adaptation

Longitudinal multi-omic data link cell-state plasticity to treatment adaptation. In 86 specimens from 49 patients, including 36 matched primary–first-recurrence pairs, recurrence showed an average shift toward mesenchymal programs, with activator protein 1 implicated in this transition and irradiation inducing related programs in vivo [22,139,140]. Recurrence also involved altered signaling among malignant, neuroglial, and immune populations [22].

This transition is not universal. A larger longitudinal study of 121 tumors from 59 patients found no malignant state consistently enriched at recurrence; most matched tumors changed their dominant state or cellular composition, but the direction varied between patients [21]. These patient-specific trajectories were associated with baseline tumor features, MGMT expression, treatment-related mutations, and coordinated microenvironmental changes [21]. Detailed eco-evolutionary recurrence patterns are considered in Section 8.

Accordingly, targeting a single transcriptional state is unlikely to eliminate the invasive compartment. Residual cells may shift toward alternative states or exploit different anatomical routes, so effective strategies may need to combine state-selective treatment with inhibition of cell-state transitions and niche-derived survival signals [22,100,121,122,129].

## 6. Spatial Organization of Niche-Dependent Glioblastoma Ecosystems

### 6.1. From Cellular States to Spatially Organized Ecosystems

Single-cell transcriptomic studies have established that GBM contains multiple malignant cell states [100]; however, tissue dissociation removes information about their anatomical localization and their interactions with neighboring cells [17,19]. Spatial transcriptomics and spatial proteomics address this limitation by mapping malignant, immune, vascular, and neural populations while preserving the architecture of the original tissue [17,18,129]. These approaches have shown that GBM cell states are not randomly distributed [17,18,19]. Instead, GBM consists of local cellular neighborhoods that are frequently dominated by a particular malignant program. Selected pairs of malignant and non-malignant cellular states also preferentially colocalize across different spatial scales [17,18]. This organization generates a recurrent, multilayered tissue architecture rather than a homogeneous mixture of transcriptionally distinct cells [17].

Hypoxia appears to be a major organizer of this spatial architecture [17,19]. Integrated spatial transcriptomic and proteomic analyses identified a five-layer spatial organization and suggested that hypoxic and necrotic regions act as long-range organizers of malignant and non-malignant cellular states [17]. Tumor regions located farther from hypoxic or necrotic foci were less spatially ordered. This observation suggests that oxygen availability may influence not only immediately adjacent cells but also the broader organization of the tumor ecosystem [17,19]. Spatial heterogeneity should therefore be viewed, at least in part, as a functional response to local microenvironmental conditions rather than solely as a static anatomical arrangement of different cellular states [17,18,141]. Spatial cellular architecture may also carry prognostic information, as higher proportions of tumor cells expressing a hypoxia-induced transcriptional program and spatial clustering of AC-like tumor cells have been associated with poorer clinical outcome [142].

### 6.2. Tumor-Core and Hypoxic–Perinecrotic Niches

The central tumor compartment encompasses highly cellular tumor regions, proliferative populations, microvascular proliferation, and spatially distinct areas of pseudopalisading necrosis [17,19,129]. Spatial analyses indicate that OPC-related and cell-cycle-associated programs are preferentially enriched in selected core regions [17,129]. In particular, OPC-like malignant cells are enriched in the tumor core and in tumor-rich regions located close to the vasculature [19,129]. By contrast, radial glial-like stem cells and AC-like populations are more abundant in neuron-rich invasive niches [129]. Even transcriptionally related tumor states may occupy separate territories. Integration of snRNA-seq with spatial transcriptomics demonstrated significant segregation of OPC-like and NPC-like tumor populations in individual GBM samples, consistent with the concept that different transcriptional states are maintained within distinct microenvironmental niches [19].

Perinecrotic regions constitute a particularly distinct spatial compartment [17,143,144]. These areas experience severe oxygen and nutrient deprivation and are enriched in hypoxia-associated, metabolic-stress, and mesenchymal-like programs [17,129,144]. Spatial transcriptomic analysis showed that perinecrotic regions displayed a more immunosuppressive profile than the broader GBM microenvironment, whereas perivascular regions exhibited comparatively pro-inflammatory signaling [19]. Experimental and spatially resolved studies further indicate that hypoxic niches recruit and sequester tumor-associated macrophages and cytotoxic T lymphocytes, promoting their reprogramming toward immunosuppressive states [101,106,143]. A distinct population of hypoxia-adapted monocyte-derived macrophages has also been localized to perinecrotic regions and associated with abnormal vascular permeability and adverse clinical outcome [101]. Immune regulation differs markedly over short anatomical distances within the same tumor. Hypoxic and perinecrotic niches may therefore support the persistence of stress-adapted tumor phenotypes while simultaneously restricting effective antitumor immunity [19,101,106,143,144,145].

The influence of hypoxia extends beyond the immediate perinecrotic compartment [17,143,146]. Its association with the global layered organization of GBM suggests that hypoxia is linked to coordinated spatial changes in malignant cell states and in surrounding immune, vascular, and brain-parenchymal populations [17,143]. Consistent with this model, a large harmonized single-cell and spatial atlas identified multiple recurrent GBM niches and found that hypoxia-dependent, highly organized tumor architectures were associated with poorer patient survival [146]. Consequently, the perinecrotic niche should not be considered simply as a non-viable region surrounding necrosis. Rather, it represents a biologically active selection compartment in which metabolic stress, hypoxia-associated and mesenchymal-like programs, vascular remodeling, and immunosuppressive signaling converge [143,144,145,146].

### 6.3. Perivascular Niches: Cellular Cooperation and Recurrence-Associated Remodeling

Perivascular regions represent a major spatial compartment of GBM [147,148,149]. Beyond supplying oxygen and nutrients, blood vessels form signaling and structural niches composed of malignant cells, endothelial cells, pericytes, fibroblast-like populations, macrophages, and extracellular matrix components [104,147,148,149]. Spatial analyses indicate that some OPC-like malignant populations are preferentially localized closer to the vasculature than to necrotic regions, potentially reflecting their higher oxygen requirements [19]. Endothelial-derived signals may additionally stabilize selected malignant phenotypes. In particular, endothelial-secreted Endocan has been shown to activate platelet-derived growth factor receptor α (PDGFRα), promote proliferative, migratory, and angiogenic properties, and confer radioprotection in experimental GBM models [103]. The vascular compartment may selectively maintain particular malignant programs rather than supporting all tumor cells equally [19,103,148].

Vascular cell populations are not uniformly tumor-promoting. Experimental depletion of pericytes caused endothelial remodeling, recruitment of immunosuppressive perivascular macrophages, increased tumor-cell invasion, and accelerated tumor progression, indicating that pericytes may exert context-dependent tumor-restraining functions [104]. The biological effects of the perivascular niche therefore depend not only on the presence of blood vessels but also on the functional state and integrity of endothelial, mural, immune, and stromal populations [104,147].

The architecture of this niche appears to change during tumor evolution. Analysis of matched primary and recurrent GBM specimens revealed a structured perivascular organization in recurrent tumors, characterized by collagen-rich ECM deposition and enrichment of CD163-positive immunosuppressive macrophages [149]. Single-cell spatial transcriptomics further identified recurrence-associated interactions among endothelial cells, perivascular fibroblast-like cells, and spatially distinct macrophage populations [149]. Independent spatial and multi-omic analyses identified enrichment of cancer-associated fibroblasts and collagen-associated extracellular matrix programs in recurrent and temozolomide-resistant GBM. These analyses also implicated tenascin-C- and filamin-C-associated endothelial-to-mesenchymal transition in the development of a fibrotic microenvironment [150]. Recurrence may involve co-evolution of malignant populations and their local tissue environment rather than clonal selection alone. Perivascular remodeling may create fibrotic, angiocrine, and immunosuppressive conditions that facilitate tumor-cell persistence and treatment resistance [103,149,150].

A high-resolution spatial atlas published in 2026 expanded this model by identifying previously undercharacterized endothelial, perivascular, and fibroblast-like states and mapping their spatial associations with malignant and immune populations [147]. The same study identified a distinct oligodendrocyte population designated Oligo_2_3_2, enriched in the tumor core and in vascular- and immune-associated regions [147]. This population exhibited an immune-activated, non-myelinating phenotype and was spatially associated with mesenchymal tumor cells and immunosuppressive macrophages [147]. Its transcriptional signature was associated with poorer overall survival in large glioma cohorts and showed increased expression in recurrent tumors [147]. Vascular niches may include not only malignant and vascular cells but also disease-associated neural-lineage populations that may contribute to the biological and prognostic heterogeneity of the GBM microenvironment [147].

### 6.4. Leading-Edge and Neuron-Rich Invasive Niches

Spatial omics has been particularly informative at the leading edge, where sparse malignant cells are interspersed with relatively preserved brain parenchyma [17,147,151,152]. Unlike the densely cellular tumor core, this compartment contains resident neurons, oligodendrocytes, astrocytes, microglia, and pre-existing vascular structures, forming a complex tumor–brain interface [17,147,148,151]. Spatial profiling demonstrated that OPC-like malignant cells were enriched in the tumor core, whereas radial glial-like stem-like populations were preferentially localized to neuron-rich invasive niches in both GBM and diffuse midline glioma (DMG) [129]. These radial glial-like populations expressed developmental and migration-associated programs and transcriptionally overlapped with subsets of astrocyte-like and mesenchymal-like malignant cells [127,129].

Ren et al. [129] mapped a trajectory from tumor core to neuron-rich infiltrated tissue in a cerebellar diffuse midline glioma (DMG) specimen. Core regions were enriched for OPC-lineage and proliferation-associated genes, whereas infiltrated neural tissue expressed radial glial, migration, synapse, axonogenesis, ECM, and inflammatory programs [129]. Although radial glial-like enrichment was also observed across GBM and DMG samples, the detailed spatial trajectory was derived from a single DMG specimen. Its transferability to adult IDH-wild-type GBM therefore requires direct validation [129].

FAM20C emerged from this analysis as a candidate regulator of radial glial-like growth in neuron-rich tissue [129]. FAM20C depletion impaired invasive growth in an orthotopic human neural stem cell-derived DMG model, with weaker effects in neuron-poor locations [129]. Because the functional validation was performed in DMG rather than adult GBM, FAM20C should be considered a hypothesis-generating, context-dependent vulnerability rather than a validated target in adult IDH-wild-type GBM. Direct validation in adult GBM models and human tissue is required [129].

Neural activity can also directly modify invasive behavior. Neuron-to-glioma synaptic input evokes calcium signals in GBM cells, and Venkataramani et al. [107] showed that neuronal activity promotes de novo tumor-microtube formation and increases invasion speed; synaptically connected invasive cells were enriched for neuronal and neural-progenitor-like states. The neuron-rich PBZ should therefore be viewed as an active electrophysiological niche, not only as a structural substrate for migration.

Independent spatial analyses of human grade 4 gliomas showed that transcriptional differences observed between mesenchymal- and proneural-classified tumor cores became less pronounced after malignant cells entered the surrounding brain tissue [151]. Malignant cells in infiltrated regions instead showed increased expression of AC-like, NPC-like, and OPC-like programs, together with gene modules associated with neurodevelopment, glial-cell differentiation, Notch signaling, and synaptic processes [151]. This convergence is consistent with the neural microenvironment promoting partially shared adaptive programs in tumor cells derived from molecularly distinct cores [107,148,151]. Accordingly, the leading edge may favor properties required for communication with resident brain cells, migration along pre-existing neural structures, and survival at low tumor-cell density.

However, this convergence should not be interpreted as evidence for a single universal invasive state. A multimodal analysis published in 2026 distinguished local and distal invasion programs: models capable of distal spread were enriched in OPC-like cells and preferentially followed peri-axonal white-matter routes, whereas models displaying predominantly local invasion were enriched in MES-like cells and favored perivascular routes [122]. Moreover, the transcriptional programs expressed during active invasion were distinct from the baseline cell-state compositions associated with a model’s general invasion propensity [122].

Single-cell spatial profiling has additionally identified region-specific extracellular matrix programs within leading-edge and infiltrating-tumor compartments [152]. In three patient-matched comparisons of GBM and non-neoplastic brain, *SEMA3E*, *COL8A1*, and *IGFBP2* were elevated in combined leading-edge and infiltrated-tumor regions, together with recurrent enrichment of additional matrisome-associated genes such as *FREM2*, *ANXA2*, and *GPC4* [152]. In a tumor containing clearly separable adjacent leading-edge and infiltrated-tissue regions, more detailed mapping distinguished astroglial programs involving *GFAP* and *AQP4* in the infiltrated brain from *IGFBP2*- and *LGALS1*-enriched programs at the leading edge [152]. *ANXA1* transcript abundance was additionally increased in more extensively infiltrated leading-edge regions, and higher annexin A1 protein expression was confirmed in independent GBM specimens [152]. The invasive front is internally heterogeneous. Rather than forming a uniform border, it contains spatially distinct astroglial, tumor-associated, and extracellular-matrix-remodeling zones [122,147,148,152].

The major spatial niches identified within glioblastoma and its invasive margin, together with their dominant cellular components and functional consequences, are summarized in Table 4.

### 6.5. Therapeutic Implications of Niche-Dependent Plasticity

Spatial studies collectively show that malignant-cell programs are coupled to anatomical context: hypoxia-associated and mesenchymal programs cluster around necrotic regions, vascular niches organize tumor–stromal interactions, and neurodevelopmental programs are enriched at neuron-rich invasive fronts [19,122,149,151]. These arrangements are dynamic, and treatment can alter both malignant-state composition and the surrounding microenvironment [21,22].The therapeutic implication is specific: a target measured in the tumor core may be absent or less relevant at the invasive edge, while slowly cycling or niche-adapted cells may escape therapies directed mainly at proliferating tumor bulk [101,122,151]. Spatial profiling can therefore help define where resistant populations reside and which local dependencies should be tested. The clinical implementation of these principles is addressed in Section 9.

## 7. The Tumor Microenvironment Protects Residual Disease

### 7.1. Therapy-Induced Remodeling Converts the Residual Tumor Bed into a Protective Niche

The microenvironment that supports residual GBM is not a static remnant of the untreated tumor. Surgery, radiotherapy, and microenvironment-directed treatments produce tissue injury and can trigger inflammatory, vascular, senescence-associated, and ECM-remodeling responses [154,155]. Although these responses accompany cytoreduction and repair, they may also create conditions that favor survival and recovery of residual malignant cells [105,156].

Longitudinal profiling of the post-surgical microenvironment has demonstrated transient blood–brain barrier disruption and time-dependent reorganization of immune populations around the resection cavity, supporting the concept that surgery creates an evolving biological niche rather than a static residual tumor bed [153]. Surgical resection may additionally induce microglia/macrophage infiltration, angiogenesis, and the production of growth factors such as pleiotrophin, which promotes self-renewal and proliferation of residual GBM stem-like cells [155].

Radiotherapy may similarly create a senescence-associated supportive niche. Irradiation-induced senescent astrocytes can produce a senescence-associated secretory phenotype, enhance chemokine signaling and promote the recruitment of inflammatory myeloid cells, thereby facilitating subsequent glioma regrowth [156,157]. Hyperplexed spatial imaging in a preclinical GBM model further demonstrated extensive reorganization of immune populations and tissue architecture following focal irradiation [154]. Notably, spatially defined, survival-promoting cellular neighborhoods emerged as early as seven days after irradiation, indicating that treatment can rapidly reshape the tumor ecosystem before radiographic recurrence becomes apparent [154].

Fibrotic niches provide a clear example of treatment-induced protection. In recurrent GBM models, resistance to CSF-1R inhibition was associated with fibrotic scars that encapsulated surviving tumor cells, promoted dormancy, and restricted immune surveillance [105]. Similar scars were observed after radiotherapy and partial resection in preclinical models and in human recurrent GBM specimens [105]. TGF-β-dependent activation of perivascular fibroblast-like cells contributed to this response, and combined inhibition of fibrosis-associated TGF-β signaling and neuroinflammation reduced scar formation and improved CSF-1R-directed treatment in preclinical models [105]. Thus, therapy can generate a tissue-repair program that shelters residual disease rather than merely selecting resistant tumor cells.

### 7.2. Myeloid Cells Couple Immunosuppression to Vascular and Fibrotic Protection

Tumor-associated microglia and macrophages can protect residual GBM through immunosuppression, trophic and angiogenic signaling, and ECM remodeling [158]. Single-cell and spatial analyses indicate that these functional programs can extend across conventional microglial and monocyte-derived identities. The GBM myeloid compartment is therefore better represented as a continuum of context-dependent states than by a binary M1/M2 classification [158].

Spatially resolved single-cell analyses identified a distinct monocyte-derived macrophage population adapted to the hypoxic perinecrotic niche [101]. These hypoxia-associated tumor-associated macrophages expressed and secreted adrenomedullin, which destabilized vascular endothelial cadherin-based endothelial adherens junctions and promoted the formation of hyperpermeable, structurally abnormal tumor vessels [101]. Despite their increased permeability, these vessels exhibited impaired perfusion and hampered effective drug delivery in GBM xenografts [101]. Genetic ablation or pharmacological blockade of macrophage-derived adrenomedullin restored vascular integrity, increased the intratumoral concentration of dabrafenib, and improved therapeutic efficacy, directly linking a niche-specific myeloid program to treatment resistance [101]. Wang et al. [101] additionally associated increased abundance of hypoxia-associated tumor-associated macrophages and adrenomedullin expression with vascular hyperpermeability and poorer clinical outcome.

Clinical translation of CSF-1R-directed myeloid targeting has nevertheless been disappointing. The brain-penetrant CSF-1R/KIT inhibitor PLX3397, also known as pexidartinib, achieved intratumoral concentrations predicted to inhibit CSF-1R and produced measurable peripheral pharmacodynamic effects in a phase II trial involving 37 patients with recurrent GBM [159]. However, six-month progression-free survival was only 8.6%, no objective responses were observed, and intratumoral pharmacodynamic effects were weaker than expected from preclinical models [159]. Moreover, drug distribution within adjacent non-enhancing and infiltrative tumor regions was not comprehensively established [159].

The limited clinical activity of CSF-1R inhibition does not make myeloid cells irrelevant targets. Instead, it illustrates functional heterogeneity, incomplete modulation across spatial compartments, and compensatory tissue responses [154,159]. In preclinical models, prolonged CSF-1R inhibition generated TGF-β- and neuroinflammation-dependent fibrotic niches that protected surviving cells; combined suppression of these responses reduced scarring and improved treatment efficacy [105]. Myeloid-directed strategies may therefore need to preserve antitumor lymphocyte activity while also limiting vascular dysfunction and fibrosis, rather than targeting one myeloid state in isolation [105,158,159].

### 7.3. ECM–Receptor Signaling Couples Adhesion, Stemness, and Resistance to Cell Death

The extracellular matrix protects residual GBM cells not only by providing a structural substrate for invasion but also by transmitting anti-apoptotic and stemness-supporting signals. Hyaluronan is a major structural and bioactive component of the brain extracellular matrix and interacts with CD44, a receptor associated with mesenchymal differentiation, invasive behavior, stem-like properties, and treatment resistance in GBM [74,160]. Within the perivascular niche, osteopontin activates CD44 signaling in adjacent tumor cells, enhancing stem-like properties and resistance to irradiation [74]. These effects involve γ-secretase-dependent release of the CD44 intracellular domain and subsequent CREB-binding protein (CBP)/p300-dependent enhancement of HIF-2α activity [74]. TGF-β signaling further reinforces this phenotype by maintaining a CD44-high/ID1-high glioma-initiating cell population with enhanced self-renewal and tumor-forming capacity [161]. Inhibition of TGF-β signaling reduced this population and impaired its capacity to reinitiate tumors in secondary recipient mice [161]. These observations link perivascular and stromal signaling to molecular programs capable of maintaining tumor-propagating cells after therapy.

Hyaluronan–CD44 signaling can also cooperate with integrin-dependent adhesion. In brain-mimetic three-dimensional hydrogel matrices containing patient-derived GBM cells, simultaneous engagement of CD44 by hyaluronan and integrin αv by Arg-Gly-Asp (RGD)-containing ligands activated Src-dependent survival signaling and suppressed the expression of pro-apoptotic BCL-2-family members, including PUMA, BAX, and BAK [160]. This cooperative signaling increased resistance to the alkylating agents temozolomide and carmustine [160]. Reducing matrix hyaluronan content or knocking down CD44 increased chemotherapy-induced apoptosis, whereas disruption of integrin–RGD binding with cilengitide enhanced the pro-apoptotic effect of chemotherapy in this experimental system [160]. These findings provide a direct mechanistic explanation for why GBM cells embedded within an appropriate three-dimensional matrix may tolerate cytotoxic exposure more effectively than cells studied in conventional two-dimensional or matrix-poor culture systems.

More recent three-dimensional modeling further links hyaluronan-rich matrices to mechanotransduction and stem-like cell programs. In scaffolds functionalized with hyaluronan, hyaluronan promoted YAP/TAZ nuclear localization and contributed to HA–CD44–CXCR4-dependent regulation of YAP/HIF signaling, hypoxic adaptation, and the localization of stemness-associated factors such as OCT4 and SOX2 [162]. The biological effects of hyaluronan depend not only on ligand–receptor binding but also on the mechanical and hypoxic properties of the surrounding matrix [162].

Integrins provide an additional link between treatment exposure and adaptive survival. Irradiation induced a dose-dependent increase in functional β1 and β3 integrins across four tested GBM cell lines and strengthened adhesion to fibronectin- and basement-membrane-like substrates [163]. However, the radioprotective effect of matrix attachment was cell-line-dependent and was clearly observed in A-172 cells but not in the other three lines [163]. Integrin blockade strongly reduced adhesion and invasion and altered the expression and gelatinolytic activity of MMP-2, whereas the effect of MMP-2/MMP-9 inhibition on invasion varied between cellular models [163]. These findings support a functional coupling between integrin-mediated adhesion, matrix-dependent survival, and metalloproteinase activity but also demonstrate that these relationships are heterogeneous rather than universal across GBM cells.

Recent spatial profiling confirms that ECM signaling is regionally organized rather than uniformly distributed. Single-cell spatial mapping of nearly 400 matrisome genes identified distinct ECM-expression programs in malignant cells, vascular endothelial cells, and microglia/macrophages [152]. Region-specific ligand–receptor networks were observed across cellular-tumor, microvascular-proliferation, and pseudopalisading/hypoxic compartments, including communication programs associated with *SPP1* and *FN1* expression between malignant and stromal populations [152]. In the same study, additional region-specific matrisome programs were identified at the leading edge and within tumor-infiltrated brain [152]. ECM composition, cellular origin, and receptor availability vary substantially over short anatomical distances within GBM.

Separate functional work identified a RUNX1–NPM1–H3K4me3 regulatory mechanism that maintained chromatin accessibility and facilitated FOSL2-dependent transcription of *FN1*, *COL4A1*, and *LUM* [164]. The resulting ECM-associated transcriptional signature was enriched in mesenchymal GBM, associated with poorer survival, and correlated with increased macrophage infiltration and an immunosuppressive tumor microenvironment [164]. The spatial and mechanistic evidence suggests that ECM remodeling integrates adhesive resistance, stemness, invasion, vascular signaling, and immune regulation rather than acting through a single matrix molecule or receptor.

The negative phase III CENTRIC trial provides an important clinical caution. Adding the selective αvβ3/αvβ5 integrin inhibitor cilengitide to standard temozolomide chemoradiotherapy did not improve outcomes in 545 patients with newly diagnosed *MGMT*-promoter-methylated GBM [165]. Median overall survival was 26.3 months in both treatment groups, with a hazard ratio of 1.02, and no predefined clinical subgroup derived a significant benefit [165].

This failure should not be interpreted as evidence that integrin-mediated adhesion is biologically irrelevant. Rather, it demonstrates that inhibition of αvβ3 and αvβ5 alone was insufficient to disrupt the broader and highly redundant ECM-signaling network. Plausible explanations include compensation by other integrins and adhesion receptors, spatial variation in ligand abundance, simultaneous CD44- and growth-factor-receptor signaling, incomplete disruption of established matrix niches, and the importance of treatment timing [152,160,165]. These explanations remain mechanistic interpretations rather than conclusions directly demonstrated by the CENTRIC trial.

### 7.4. Hypoxia-Associated CXCL12–CXCR4 Signaling Supports Vascular Recovery After Radiotherapy

Hypoxia is an important mechanism linking radiation-induced vascular injury with microenvironmental rescue. In experimental GBM, irradiation reduced endothelial-cell abundance and tumor perfusion while increasing intratumoral hypoxia and HIF-1 activity [166]. HIF-1-dependent induction of CXCL12, also known as stromal cell-derived factor 1, promoted CXCR4-dependent recruitment and retention of bone-marrow-derived cells within the irradiated tumor [166]. Most of the recruited cells were CD11b-positive myelomonocytic cells rather than endothelial progenitor cells and were spatially associated with, but did not substantially incorporate into, the vascular endothelium [166]. These cells supported the restoration of functional tumor perfusion and enabled the regrowth of surviving malignant cells.

Pharmacological inhibition of HIF-1 or disruption of CXCL12–CXCR4 signaling with AMD3100 (plerixafor) prevented the post-radiation influx of bone-marrow-derived myeloid cells, inhibited restoration of tumor perfusion, and abrogated recurrence in intracranial xenograft models [166]. Similar effects were observed with a CXCR4-neutralizing antibody and with experimental depletion of monocytes and macrophages [166]. By contrast, VEGFR2 inhibition delayed regrowth but did not prevent recurrence and was less effective than CXCR4 blockade in suppressing the recovery of tumor perfusion [166]. Vasculogenesis can function as an important compensatory pathway when local angiogenesis has been impaired by irradiation.

This distinction is particularly relevant to residual disease. In the model used by Kioi et al. [166], untreated intracranial tumors relied predominantly on local angiogenesis, whereas post-irradiation regrowth became dependent on bone-marrow-derived cell-supported vasculogenesis. Beyond its role in vasculogenesis, CXCL12–CXCR4 signaling can promote GBM-cell invasion and treatment resistance and contribute to the recruitment of immunosuppressive myeloid cells. CXCR4 inhibition reduced the migration of glioma stem-like cells toward endothelial and subventricular-zone niches, whereas genetic CXCR4 knockdown decreased perivascular invasion and increased radiation-induced apoptosis in orthotopic glioma models [137,167]. In separate preclinical models, blockade of CXCL12–CXCR4 signaling reduced the intratumoral accumulation of CXCR4-positive monocytic myeloid-derived suppressor cells [138]. However, the relationship between hypoxia and CXCL12 should not be viewed as universally linear. Recent spatial analyses of human GBM showed a strong association between hypoxia and *VEGFA*, whereas *CXCL12* expression was only weakly associated with hypoxia and was distributed among endothelial cells, pericytes, macrophages/microglia, and malignant cells [168]. *CXCL12* was particularly abundant in vascular and infiltrative compartments, suggesting that its expression is controlled by both hypoxic and non-hypoxic, cell-type-specific mechanisms [168].

Early clinical studies provide preliminary but non-definitive support for therapeutic targeting of this pathway. In the non-randomized phase I/II Macrophage Exclusion after Radiation Therapy trial, 29 patients with newly diagnosed GBM received a continuous four-week infusion of the CXCR4 inhibitor plerixafor beginning on day 35 of concurrent chemoradiotherapy. The treatment was well tolerated, and median progression-free and overall survival were 14.5 and 21.3 months, respectively [169]. First progression occurred within the irradiation field in 47% of evaluable patients, accompanied by a sustained reduction in relative cerebral blood volume within the treated field [169]. Although these imaging and recurrence patterns were consistent with inhibition of local post-radiation revascularization, the small, single-arm design, molecular heterogeneity of the cohort, and comparison with non-randomized controls preclude definitive conclusions regarding efficacy [169].

The dose-escalation part of the phase I/II GLORIA trial subsequently evaluated radiotherapy combined with the CXCL12-neutralizing L-RNA aptamer olaptesed pegol, also known as NOX-A12, in ten patients with incompletely resected or biopsied, newly diagnosed, IDH-wild-type, *MGMT*-promoter-unmethylated GBM [170]. No dose-limiting toxicities or treatment-related deaths were observed, and 600 mg per week was selected as the recommended phase II dose [170]. Nine patients showed a reduction in MRI lesion size at one or more assessment time points, while four met modified Response Assessment in Neuro-Oncology (mRANO) criteria for partial response [170]. Nine patients also exhibited a reduction in relative cerebral blood volume at best response. Median progression-free survival was 174 days, six-month progression-free survival was 40%, and median overall survival was 389 days [170]. These observations demonstrate feasibility and provide an efficacy signal but require cautious interpretation because of the very small cohort, absence of randomization, and exploratory nature of several imaging and biomarker analyses.

A subsequent six-patient GLORIA expansion arm combined radiotherapy and NOX-A12 with bevacizumab to target complementary CXCL12-dependent vasculogenesis and VEGF-dependent angiogenesis [168]. The combination was well tolerated, with no dose-limiting toxicities or treatment-related deaths [168]. Confirmed best responses included one complete response, four partial responses, and one case of stable disease, corresponding to an objective response rate of 83.3% [168]. Median progression-free and overall survival from treatment initiation were 9.1 and 19.9 months, respectively, and two patients survived for more than two years [168]. Outcomes appeared more favorable than in the preceding radiotherapy plus NOX-A12 cohort, but this comparison was post hoc and involved only six patients [168]. The study therefore provides proof of principle for simultaneous targeting of complementary revascularization pathways but does not establish superiority over bevacizumab alone or over current standard treatment. Prospective randomized studies are required to determine whether dual inhibition of CXCL12 and VEGF produces a reproducible survival benefit.

### 7.5. Why Single-Compartment Targeting Is Insufficient

The available clinical evidence indicates that inhibition of a single microenvironmental pathway is generally insufficient to produce durable disease control in GBM. In recurrent GBM, the brain-penetrant CSF-1R/KIT inhibitor PLX3397 achieved measurable intratumoral exposure but yielded a six-month progression-free survival rate of only 8.6%, with no objective responses [159]. In newly diagnosed *MGMT*-promoter-methylated GBM, the addition of the αvβ3/αvβ5 integrin inhibitor cilengitide to standard chemoradiotherapy did not improve overall survival in the phase III CENTRIC trial [165]. Similarly, the TGF-β receptor inhibitor galunisertib, administered alone or in combination with lomustine, failed to improve survival in recurrent GBM [171].

These negative trials should not be interpreted as evidence that macrophages, integrins, or TGF-β are biologically irrelevant. Rather, they demonstrate that the protective functions of the GBM microenvironment are distributed across partially redundant cellular and molecular systems. Residual malignant cells may compensate for inhibition of one pathway by transitioning toward an alternative transcriptional state, recruiting different immune or stromal populations, or relocating to another vascular or extracellular-matrix-supported niche [21,22,105]. In addition, target expression and drug exposure may differ substantially between the CET, the invasive margin, and more distant infiltrated brain tissue.

The emergence of TGF-β- and neuroinflammation-dependent fibrotic niches after prolonged CSF-1R inhibition provides direct evidence of such compensatory remodeling. Rather than eliminating microenvironmental support, suppression of the myeloid compartment promoted the formation of fibrotic structures that encapsulated surviving malignant cells, restricted immune surveillance, and facilitated their persistence [105]. Failure of single-compartment strategies may reflect ecological adaptation of the residual tumor–microenvironment system rather than inadequate biological relevance of the targeted pathway.

The GBM microenvironment functions as an adaptive network rather than as a collection of independent therapeutic targets. Effective disruption of residual disease will therefore require strategies that account for compensatory interactions among malignant cells, immune populations, vascular structures, and extracellular matrix components. The design of such multi-compartment and spatially coordinated approaches is discussed in Section 9.

The principal forms of treatment-induced microenvironmental remodeling that may protect residual disease and promote recurrence are summarized in Table 5.

## 8. Recurrence Is a Spatial and Ecological Process

### 8.1. Recurrence Extends Beyond a Linear Model of Clonal Evolution

Glioblastoma recurrence has often been conceptualized through a predominantly linear model in which treatment eliminates sensitive cells and permits the expansion of a genetically resistant clone. However, longitudinal genomic studies indicate that this framework captures only part of the evolutionary process [173,174].

The Glioma Longitudinal Analysis Consortium (GLASS) analyzed temporally separated tumors from 222 adults with diffuse glioma and identified multiple evolutionary trajectories, broad preservation of truncal drivers, and little evidence for a universal set of recurrence-specific driver alterations [173]. A subsequent GLASS analysis of 304 patients integrated longitudinal DNA and RNA data and showed that progression reflects both genetic evolution and changes in cellular composition [174]. In IDH-wild-type disease, recurrence was associated with more invasive and neuronal-signaling programs, whereas mesenchymal transitions were linked to malignant–myeloid interactions [174].

An independent longitudinal proteogenomic analysis included 123 matched GBM pairs. Recurrent tumors showed a transition from highly proliferative programs at diagnosis toward neuronal and synaptogenic states, accompanied by activation of WNT/planar cell polarity and BRAF signaling [175].

These findings argue against recurrence being driven by the acquisition of a universally advantageous mutation. Instead, they support a model in which therapy acts on a heterogeneous malignant population embedded within a changing tissue ecosystem.

Single-cell studies reinforce this heterogeneity. In 86 longitudinal specimens from 49 patients, recurrence showed an average mesenchymal shift with altered malignant–neuroglial–immune signaling [22]. In a larger series of 121 tumors from 59 patients, however, no malignant state was consistently enriched at recurrence; most matched pairs changed dominant state or composition, but in different directions [21]. Recurrent GBM is therefore better described as a set of patient-specific evolutionary trajectories than a single endpoint [21,22].

Genetic evolution nevertheless remains relevant. Temozolomide-associated hypermutation has been documented in a subset of recurrent gliomas following exposure to alkylating therapy [172,173], whereas radiotherapy has been associated with characteristic small- and large-deletion signatures [176]. Recurrence-associated copy-number alterations also occur, but they are context dependent. For example, acquired *CDKN2A* loss, additional cell-cycle alterations, and increased aneuploidy were enriched in IDH-mutant, 1p/19q-intact glioma rather than uniformly across recurrent GBM [173,174]. These genomic events are important in selected patients but do not by themselves explain the broad diversity of recurrence-associated phenotypes [173,176]. Genomic selection, phenotypic plasticity, and microenvironmental remodeling should therefore be viewed as complementary rather than competing mechanisms [21,22,174].

### 8.2. Spatially Heterogeneous Treatment Pressure Creates Multiple Potential Founders of Recurrence

Treatment pressure is spatially non-uniform. Surgery removes the CET but leaves distinct malignant populations in non-enhancing brain [13,177]. Radiotherapy creates a spatial dose gradient, and recurrence can occur within high-dose regions or outside the principal field [178]. Paired spatial profiling shows that treatment also reorganizes vascular, glial, and immune neighborhoods [20]. Drug exposure varies by region: image-registered human samples show differences between enhancing and non-enhancing tissue [179], and a phase 0 ribociclib study measured lower unbound concentrations in non-enhancing recurrent GBM than in enhancing tissue [25]. Residual populations therefore experience different combinations of treatment pressure and niche protection.

This spatial heterogeneity provides several potential founder populations from which recurrence may arise. The longitudinal cohort included 38 patients with GBM. Mutation-retention analysis was possible in 22 evaluable primary–recurrent tumor pairs and showed a strong association between recurrence location and evolutionary divergence [180]. All seven distant recurrences retained fewer than half of the mutations detected in the corresponding primary tumor. Their mean retention rate was approximately 25%. By contrast, 12 of 15 local recurrences retained more than half of the primary-tumor mutations, with a mean retention rate of approximately 70% [180]. Distant recurrences frequently displayed branched evolution and substantial divergence in key GBM driver alterations. Local recurrences generally showed greater genetic continuity with the primary lesion [180]. These findings are consistent with different origins of local and distant recurrence. Some local recurrences may arise from residual populations near the resection cavity. By contrast, distant recurrences may originate from earlier-diverging populations that disseminated before diagnosis and remained spatially separated from the dominant primary-tumor clone [180].

Three-dimensional whole-tumor profiling further demonstrates why a single diagnostic specimen may fail to identify the population that eventually contributes to recurrence. Three-dimensional profiling included 103 spatially registered samples from ten newly diagnosed, treatment-naïve, IDH-wild-type GBMs. The analysis identified tumor-wide alterations reflecting early evolutionary events. It also revealed genetic subclones and tumor- or microenvironment-associated programs restricted to the tumor core, periphery, or contrast-enhancing compartment [181]. Spatially restricted alterations included region-specific oncogene amplifications, tumor-suppressor deletions, structural variants, chromatin states, and transcriptional programs [181]. Because the study analyzed untreated primary tumors rather than matched recurrences, it does not directly identify recurrence-founding populations. Nevertheless, the study provides a mechanistic explanation for an apparent change in tumor identity at recurrence. Such a change may partly reflect expansion of a pre-existing population that was absent or underrepresented in the original diagnostic specimen [180,181].

Accordingly, recurrence should not be conceptualized simply as the re-emergence of the average primary-tumor cell. It is more plausibly generated by one or several residual populations whose fitness is context-dependent [21,180,181]. The clone or cellular state that dominates the untreated tumor core may not be the population best equipped to survive within irradiated white matter, a remodeled perivascular niche, or the postoperative resection margin [151,181]. A recent multicenter study analyzed 390 paired MRI examinations from patients with CNS WHO grade 4 glioma. Primary-tumor composition, anatomical location, and time to progression were associated with subsequent spatial recurrence trajectories. These findings support further investigation of individualized radiotherapy margins [182].

### 8.3. Malignant Cells and the Tumor Microenvironment Co-Evolve During Recurrence

The ecological model of recurrence predicts that changes in malignant cells should be accompanied by coordinated changes in the surrounding tissue. This prediction is supported by several longitudinal datasets. In the GLASS cohort, recurrent IDH-wild-type malignant cells showed increased neuronal-signaling programs and altered tumor–neural interactions. Mesenchymal transitions were associated with a distinct myeloid-cell state and reciprocal ligand–receptor signaling between malignant and myeloid populations [174].

Similarly, longitudinal single-cell studies demonstrated that primary-to-recurrent evolution involves coordinated changes in malignant-cell states and in the composition and functional programs of non-malignant neuroglial and immune populations [21,22]. The Glioblastoma Cellular Analysis of Resistance and Evolution (CARE) cohort included 59 patients. Its most consistent longitudinal finding was a lower relative malignant-cell fraction at recurrence, accompanied by increased proportions of glial and neuronal cells within the tumor microenvironment (TME) [21]. This compositional trajectory was observed in approximately 66% of matched cases and was independently reproduced through reanalysis of a separate longitudinal single-nucleus RNA-sequencing cohort [21,22]. Changes in malignant-state abundance also mirrored changes in TME composition and broader tumor-level expression programs, supporting the concept of an interdependent evolving ecosystem rather than two independently changing compartments [21].

Spatially resolved longitudinal evidence further supports this model. Imaging mass cytometry was applied to paired primary and locally recurrent IDH-wild-type GBMs. Recurrent tumors showed reduced vascular-cell abundance, increased nonmalignant brain-cell populations, and altered spatial interactions among malignant cells, astrocytes, oligodendrocytes, and vascular cells [20]. These findings suggest that recurrence-associated evolution includes not only changes in cell abundance but also reorganization of the cellular neighborhoods in which residual malignant cells reside. Because this study included only five patients; however, its spatial findings should be considered exploratory rather than universally representative [20].

Immune evolution provides another example of tumor–microenvironmental co-dependence. Single-cell profiling was performed in an epidermal growth factor receptor-driven, immunocompetent mouse model of GBM. Early tumors contained pro-inflammatory microglial populations, whereas late-stage tumors were enriched in anti-inflammatory macrophages, neutrophils, and tumor-supportive myeloid-derived suppressor cells (MDSCs) [183]. A corresponding shift in the microglia-to-macrophage balance was observed in cross-sectional human specimens from low-grade glioma and GBM [183]. However, these samples did not constitute a longitudinal primary–recurrent cohort.

Treatment altered this immune composition in the same experimental model. Temozolomide reduced the accumulation of selected MDSC populations in this model. Combined temozolomide and irradiation increased intratumoral granzyme B–positive CD8+ cytotoxic T cells but also expanded immunosuppressive CD4+ regulatory T cells (Tregs) [183]. Therapy did not produce a uniformly stimulatory or suppressive immune response; instead, it generated concurrent antitumor and tumor-protective immune changes [183]. Because these treatment experiments were performed primarily in mice, their direct relevance to human recurrent GBM requires confirmation in prospective longitudinal cohorts.

Human longitudinal data nevertheless indicate that the myeloid compartment is also remodeled at recurrence. Multidimensional analysis of matched primary and recurrent IDH-wild-type GBMs identified activation of microglial programs and enrichment of lymphoid populations in selected rapidly recurring tumors. Expression of Fc gamma receptors and complement-associated genes at recurrence was inversely associated with time to relapse [184]. The study was based on a relatively small cohort, and spatial analyses were available only for patient subsets. Nevertheless, it provides human evidence that immune-state remodeling may be associated with the clinical tempo of recurrence [184].

Transcriptomic analysis of paired primary and recurrent IDH-wild-type GBMs showed comparatively limited longitudinal changes in many hallmark GBM genes. Nevertheless, a subset of tumors shifted toward mesenchymal transcriptional programs [185]. By contrast, recurrence was accompanied by substantial changes in inferred tissue composition, including reduced tumor purity, increased neuronal and oligodendroglial signals, increased tumor-associated macrophage programs, and reduced endothelial-cell markers [185]. An ECM-associated gene signature increased at recurrence and was localized predominantly to pericyte-like cells by bulk RNA sequencing, single-cell analysis, and immunohistochemistry [185]. Higher expression of this signature in recurrent tumors was associated with poorer survival following the second surgery [185].

This observation is particularly important because it indicates that clinically relevant tumor evolution can occur without a correspondingly large change in the canonical molecular identity of the malignant compartment. In some patients, a decisive evolutionary event may therefore involve reconstruction of a vascular, immune, or ECM-supported habitat rather than the emergence of a fundamentally new tumor genotype [20,184,185].

### 8.4. Recurrent GBM Follows Multiple Eco-Evolutionary Trajectories

Longitudinal studies have reported different dominant phenotypes at recurrence. A single-cell multi-omic analysis identified an average shift toward a mesenchymal-like state mediated in part by activator protein 1 signaling [22]. By contrast, integrated proteogenomic analysis of 123 longitudinal GBM pairs showed replacement of a highly proliferative diagnostic state by increased neuronal and synaptogenic programs at recurrence [175]. This neuronal transition was associated with post-translational activation of WNT/planar cell polarity signaling and BRAF kinase and was reproduced in patient-derived xenograft models [175].

These results should not necessarily be regarded as contradictory. RNA-, protein-, and phosphoproteomic measurements capture different levels of tumor biology, while bulk analyses are influenced by the proportions of malignant and non-malignant cells in each specimen. In addition, the larger longitudinal single-cell cohort demonstrated that no single trajectory dominates across all patients [21]. Some recurrent tumors become enriched in mesenchymal- or hypoxia-associated populations, others develop neuronal or developmental programs, and still others preserve a cellular composition resembling the primary tumor. The selected phenotype may depend on the cell states present at diagnosis, *MGMT* status, treatment-associated genetic damage, and the composition of the residual niche [174,175].

Spatial analysis of matched primary and recurrent human GBMs provides evidence that genetically distinct malignant populations are associated with specific tissue habitats. In recurrent tumors, extracellular matrix organization was associated with the spatial distribution of genetically distinct malignant-cell populations [149]. Recurrent perivascular niches displayed more structured ECM organization and were enriched in transcriptionally distinct immunosuppressive macrophages. Spatial transcriptomics additionally identified interactions among endothelial cells, perivascular fibroblast-like cells, and these macrophage populations [149]. The niche therefore acts as more than a passive background and may contribute to the localization and functional maintenance of particular malignant populations.

The same principle applies to treatment-associated fibrotic niches. In preclinical models, these niches can encapsulate residual cells, restrict immune surveillance, and support dormant persistence [105]. Hypoxic compartments similarly support stress-adapted malignant and myeloid states [21,101]. In ecological terms, these niches represent distinct habitats with different resources, hazards, and intercellular relationships. A phenotype that is poorly competitive in the untreated tumor core may become highly fit after treatment if the surrounding habitat has been transformed in its favor.

### 8.5. An Integrated Eco-Evolutionary Model of GBM Recurrence

An integrated model can be stated in four steps. First, the untreated tumor contains genetically and phenotypically diverse populations distributed across distinct niches [117,181]. Second, surgery removes the central bulk but leaves residual cells in the invasive margin and other protected compartments [180,181]. Third, radiotherapy and chemotherapy impose selective pressure while remodeling vascular, immune, neural, and ECM components [174,185]. Fourth, surviving populations adapt through clonal selection, reversible state transitions, and renewed interactions with the altered microenvironment [22,173].

The recurrent lesion is therefore an ecosystem reconstructed from residual malignant populations and treatment-altered host tissue [21,185]. Its composition depends on pre-treatment diversity, the location of surviving cells, regional treatment exposure, and the response of the surrounding brain. This model explains why major drivers may be retained while transcriptional, proteomic, immune, and stromal properties change substantially between diagnosis and recurrence [174,175,185].

Standard therapy does not select one optimal phenotype across all patients; instead, it modifies the fitness landscape within each individual tumor [173,174]. The resulting recurrence reflects the population best adapted to the specific combination of residual niches and treatment-associated environmental changes in that patient [117,174,186].

### 8.6. Clinical and Experimental Implications

The ecological nature of recurrence has direct implications for tissue monitoring. Molecular characterization at diagnosis should not automatically be assumed to represent recurrent disease because paired specimens can change in epigenetic class and cellular composition [23]. When re-resection or biopsy is clinically indicated and neurologically safe, recurrent tissue should be treated as a new biological specimen and re-profiled if the result could affect trial eligibility or treatment selection [23,24].

Longitudinal studies should integrate genomic and epigenomic profiling with measurements of malignant-cell states, immune composition, vascular organization, and extracellular matrix structure [14,23].

Longitudinal tissue profiling does not imply routine serial PBZ biopsy in clinically stable patients. In current human studies, the material is usually tissue obtained at the initial operation and, in a subset of patients, at clinically indicated re-resection or biopsy for suspected recurrence [14,23,24]. Elective repeated PBZ sampling is constrained by procedural risk and by the fact that a single specimen can underrepresent regional heterogeneity. When tissue is available, multiregion sampling should obtain separate neuronavigation- or image-registered specimens from anatomically distinct enhancing and non-enhancing regions rather than pooling them [13,14]. Spatial registration preserves the imaging coordinates of each specimen, allowing molecular findings to be assigned to CET, nCET/PBZ, or a recurrence habitat. This reduces—but does not eliminate—sampling bias and helps distinguish treatment-associated changes from pre-existing regional populations.

Clinical trials should also make the consequences of profiling explicit. The N2M2 umbrella trial prospectively allocated 228 patients with newly diagnosed MGMT-unmethylated GBM across molecularly matched or nonmatched subtrials [187]. The phospho-mTOR-selected temsirolimus cohort (*n* = 46) achieved a six-month progression-free survival rate of 39.1%, compared with 18.5% in the temozolomide standard-of-care group (*n* = 54), whereas the atezolizumab, asunercept, and palbociclib subtrials did not meet their efficacy endpoint [187]. Thus, prospective molecular matching is feasible, but a plausible target does not guarantee clinical benefit.

For invasive-margin trials, the target compartment and the measurement strategy should be prespecified. A PBZ-directed drug should demonstrate exposure and target modulation in non-enhancing tissue, not only in the CET [15,25,179]. Relevant imaging endpoints include change in a predefined high-risk region and mapping of local versus distant failure; overall survival remains essential because contrast enhancement can change through vascular or inflammatory effects without a proportional change in viable tumor burden [28]. Between surgical time points, serial MRI and, in selected settings, amino-acid PET can provide longitudinal spatial context, but they do not replace tissue for genomic or single-cell analysis [26,27].

**Figure 4:**
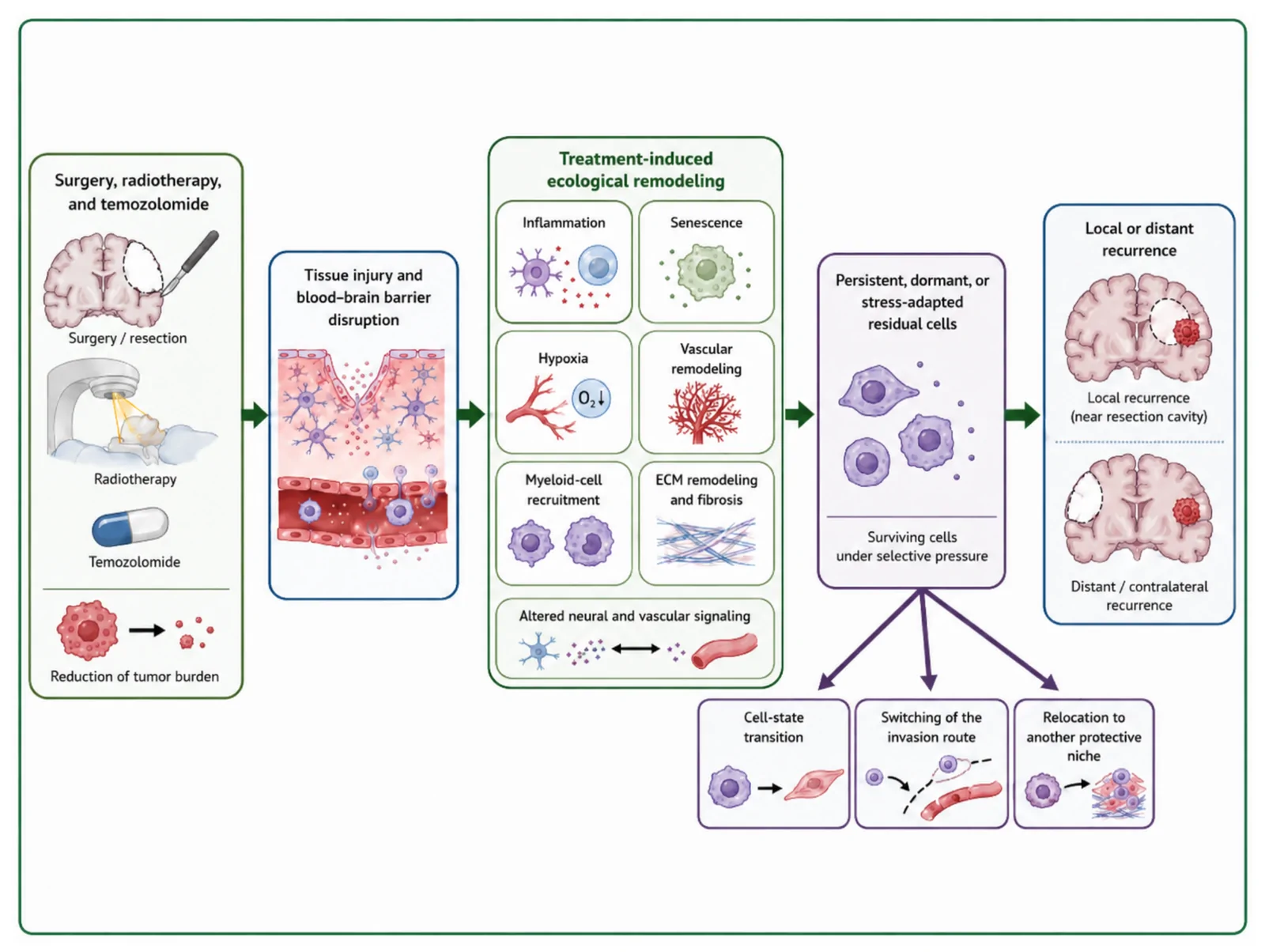
Treatment-induced ecological remodeling and glioblastoma recurrence. Surgery, radiotherapy, and temozolomide reduce tumor burden but also induce tissue injury, blood–brain barrier disruption, inflammation, senescence, hypoxia, vascular remodeling, myeloid-cell recruitment, and extracellular-matrix remodeling. The resulting selective pressure may support persistent, dormant, or stress-adapted residual cells that undergo cell-state transitions, switch invasion routes, or relocate to protective niches, ultimately contributing to local or distant recurrence. Abbreviation: ECM, extracellular matrix.

The proposed sequence linking standard treatment, microenvironmental remodeling, residual-cell adaptation, and subsequent local or distant recurrence is schematically summarized in Figure 4.

## 9. Therapeutic Implications: From Tumor-Bulk Control to Targeting Residual Invasive Disease

### 9.1. Extending the Therapeutic Target Beyond Contrast Enhancement

The central therapeutic implication of the evidence discussed above is that successful GBM treatment cannot be defined exclusively by removal or radiographic control of the CET. The clinically relevant target is the residual disease field, comprising infiltrating malignant cells, treatment-adapted cellular states, and supportive niches distributed throughout the peritumoral brain. Safe supramaximal resection of selected non-contrast-enhancing tissue has been associated with improved survival compared with resection limited to the contrast-enhancing lesion. However, the available evidence is predominantly retrospective and vulnerable to selection bias. It does not support indiscriminate resection of all fluid-attenuated inversion recovery (FLAIR)-hyperintense tissue [31]. The therapeutic objective should therefore be maximal biologically informed disease control without compromising neurological function.

Current European Society for Radiotherapy and Oncology–European Association of Neuro-Oncology (ESTRO–EANO) recommendations define the radiotherapy gross tumor volume primarily from the resection cavity and residual T1 contrast-enhancing abnormality, followed by an anatomically modified 15 mm clinical target volume margin [55]. This standardized strategy addresses microscopic extension at the population level but does not account for patient-specific variation in invasion routes, perfusion, hypoxia, white-matter connectivity, or niche composition. Recent multicenter analysis of 390 paired primary and recurrent grade 4 glioma MRIs showed that baseline tumor composition, anatomical location, and time to progression were associated with subsequent spatial recurrence trajectories [182]. These observations provide a rationale for prospective evaluation of biologically risk-adapted radiotherapy volumes or dose painting based on multiparametric MRI, amino acid positron emission tomography (PET), radiomics, and spatial biomarkers. Such approaches should currently be regarded as investigational rather than replacements for guideline-based target delineation [27,188].

### 9.2. Demonstrating Drug Exposure in Non-Contrast-Enhancing Brain

A molecularly appropriate drug cannot eliminate residual invasive disease if it fails to reach the relevant anatomical compartment. Conventional pharmacokinetic assessment based on plasma, cerebrospinal fluid, or CET tissue may substantially overestimate exposure within the non-enhancing brain. In a phase 0 study of ribociclib, mean unbound drug concentrations were 2.152 μmol/kg in enhancing tumor but only 0.560 μmol/kg in non-enhancing tissue [25]. Although target-relevant concentrations were achieved in both compartments, the approximately fourfold regional difference illustrates why pharmacokinetic and pharmacodynamic measurements should be obtained directly from image-localized non-enhancing tissue whenever feasible. Target engagement in the enhancing tumor core should not be considered sufficient evidence that an agent can treat infiltrating cells.

Focused ultrasound-mediated blood–brain barrier opening provides one strategy for increasing regional drug exposure. In a phase I trial involving 17 patients with recurrent GBM, repeated low-intensity pulsed ultrasound with microbubbles increased brain concentrations of albumin-bound paclitaxel and carboplatin by approximately 3.7-fold and 5.9-fold, respectively [189]. A subsequent phase I/II trial involving 33 patients demonstrated that repeated activation of a nine-emitter ultrasound implant could produce MRI-confirmed blood–brain barrier opening during carboplatin treatment without dose-limiting toxicity [190]. More recently, the multicenter BT008NA phase I/II trial applied transcranial microbubble-enhanced focused ultrasound specifically to periresectional, tumor-infiltrative regions during adjuvant temozolomide therapy [191]. These trials establish the feasibility of repeated, spatially targeted blood–brain barrier opening, but their single-arm or early-phase designs do not yet establish a survival benefit.

Direct local delivery—including biodegradable polymers, injectable hydrogels, intracavitary reservoirs, and convection-enhanced delivery—can achieve high concentrations near the resection cavity while limiting systemic toxicity [192]. Its principal limitation is spatial coverage: diffusion and convection are influenced by catheter position, pressure gradients, tissue anisotropy, postoperative cavities, necrosis, and heterogeneous extracellular matrix architecture [192]. Local delivery is therefore best viewed as a method for intensifying treatment within the highest-risk postoperative compartment, not as a complete solution for malignant cells distributed along white-matter tracts, perivascular routes, or distant brain regions.

### 9.3. Combining Local Postoperative Treatment with Distributed Disease Control

The resection cavity provides a rational therapeutic access point because it is adjacent to the predominant site of recurrence and can be targeted before the postoperative niche has fully matured. Locoregional cellular and viral therapies have shown that repeated treatment can be delivered directly into the tumor cavity or cerebrospinal fluid. A phase I trial evaluated locoregional IL-13Rα2-targeted chimeric antigen receptor T-cell (CAR-T) therapy in 65 patients with recurrent high-grade glioma. Stable disease or better was observed in 50% of evaluable patients. However, median overall survival in the recurrent GBM subgroup was 7.7 months, and the nonrandomized design precluded conclusions regarding comparative efficacy [193].

Multi-antigen targeting may reduce escape caused by regional antigen heterogeneity. In a 2025 phase I study of 18 patients with recurrent GBM, intracerebroventricular CAR-T cells targeting both EGFR and IL-13Rα2 produced radiographic tumor reduction in 8 of 13 patients with measurable postoperative disease [194]. Most responses were transient, indicating that broader antigen coverage can induce tumor regression but does not by itself overcome limited CAR-T-cell persistence, immunosuppression, or spatially dispersed antigen-negative populations.

A similar principle is illustrated by local oncolytic virotherapy. Intratumoral DNX-2401 followed by systemic pembrolizumab was evaluated in 49 patients with recurrent GBM. The objective response rate was 10.4% and did not meet the prespecified primary efficacy threshold, although median overall survival was 12.5 months and the 12-month survival rate was 52.7% [195]. These results suggest that local tumor disruption can generate durable benefit in a subset of patients, but local immune activation alone is unlikely to control the full spatial extent of residual disease.

An important randomized example of sustained, spatially distributed postoperative treatment is provided by tumor-treating fields. In the EF-14 trial involving 695 patients, the addition of tumor-treating fields to maintenance temozolomide prolonged median progression-free survival from 4.0 to 6.7 months and median overall survival from 16.0 to 20.9 months [196]. A subsequent systematic review and meta-analysis of seven comparative studies comprising 1430 patients reported a pooled overall-survival hazard ratio of 0.63 for tumor-treating fields plus standard treatment compared with standard treatment alone [197]. Tumor-treating fields are not a PBZ-specific or niche-selective intervention, and higher device adherence was associated with improved survival in a subgroup analysis of the EF-14 trial [198]. Nevertheless, they provide clinical evidence that sustained, non-surgical treatment applied after chemoradiotherapy can improve survival when added to standard maintenance therapy.

These findings support a combined therapeutic architecture: intensive local treatment of the recurrence-prone resection margin should be coupled with a distributed modality capable of reaching cells beyond the immediate postoperative field. Local and distributed therapies should therefore be considered complementary rather than competing strategies.

### 9.4. Designing Combinations Against Orthogonal Mechanisms of Failure

Effective combinations should not be designed by simply adding multiple agents directed against closely related pathways. A biologically rational regimen should address at least three distinct requirements: elimination of a dominant residual malignant-cell population, disruption of the niche that maintains that population, and adequate delivery of both interventions to the relevant spatial compartment. Longitudinal single-cell analyses show that no single malignant state is uniformly selected at recurrence [21,22], while experimental studies demonstrate that inhibition of one supportive compartment can induce compensatory fibrosis or recruitment of an alternative stromal population [105].

The limited clinical activity of CSF-1R inhibition, αvβ3/αvβ5 integrin inhibition, and TGF-β inhibition as single-pathway strategies illustrates the limitations of targeting one element of an adaptable ecosystem [159,165,171]. Future combinations should therefore be selected on the basis of non-redundant mechanisms and demonstrated target engagement, rather than on the independent activity of each agent in tumor-core models.

Several recent approaches illustrate this design principle. Dual targeting of CXCL12-dependent vasculogenesis and VEGF-dependent angiogenesis attempts to suppress complementary mechanisms of post-radiation vascular recovery [168]. GPNMB-directed CAR-T cells were designed to eliminate both treatment-resistant malignant cells and immunosuppressive myeloid populations expressing the same surface antigen [199]. The postoperative exosome–hydrogel system described above combined direct killing of residual malignant cells, suppression of Notch-dependent glioma-initiating-cell programs, and local immune activation [200]. Multi-antigen CAR-T-cell therapy similarly attempts to reduce escape through regional antigen loss [194]. Although these strategies remain preclinical or early phase, they provide more convincing therapeutic concepts than combinations targeting only one cell population or one signaling pathway.

Treatment sequence is likely to be as important as drug selection. The immediate postoperative period may favor local delivery before fibrotic remodeling and restoration of the blood–brain barrier, whereas inhibition of CXCL12-dependent vasculogenesis may be most relevant after radiation-induced vascular injury. Immunotherapy may require scheduling that limits corticosteroid exposure and precedes the establishment of profound local lymphocyte dysfunction [201]. Therapeutic timing should therefore be aligned with the biological state of the postoperative and post-radiation niche rather than determined solely by conventional chemotherapy cycles.

The main therapeutic strategies discussed above, their intended spatial or biological targets, and their current level of evidence are summarized in Table 6.

### 9.5. Measuring Spatial Target Engagement in Clinical Trials

Where feasible, early-phase trials should establish whether an investigational treatment reaches and modifies residual invasive disease before large-scale efficacy testing. Phase 0 and window-of-opportunity designs are particularly suitable because they allow preoperative drug exposure followed by image-localized tissue sampling and direct measurement of pharmacokinetics, target inhibition, cell-state changes, and microenvironmental responses [15]. Sampling should include both contrast-enhancing and non-enhancing regions whenever neurologically safe, because analysis of the resected tumor core alone may provide a misleading estimate of activity against residual disease [15,25].

Neoadjuvant anti-programmed cell death protein 1 (PD-1) studies illustrate both the value and limitations of this approach. An initial randomized pilot study showed that presurgical pembrolizumab induced intratumoral and systemic immune changes and suggested a survival benefit compared with exclusively adjuvant treatment [202]. However, a subsequent single-arm expansion cohort confirmed suppression of cell-cycle programs and induction of T-cell/interferon-related signatures but did not reproduce the survival advantage; pooled six-month progression-free survival was 19.5% [203]. The critical lesson is that pharmacodynamic activity is necessary but not sufficient evidence of therapeutic efficacy.

Trials directed at residual invasive disease should therefore incorporate spatially explicit endpoints. Biological endpoints should include drug concentration and target modulation in non-contrast-enhancing tissue, together with longitudinal changes in malignant and nonmalignant cell states. Clinical and imaging endpoints should include changes in high-risk regions, local versus distant failure, progression within and outside the treatment field, and corticosteroid exposure. Overall survival should remain the definitive efficacy endpoint, because contrast enhancement can be altered by vascular normalization, blood–brain barrier opening, local inflammation, or immunotherapy without proportional changes in viable tumor burden [28].

At present, no treatment selected specifically by a molecular or spatial PBZ biomarker has demonstrated an overall-survival benefit in a randomized trial. The immediate translational priority is therefore not indiscriminate escalation of treatment throughout the FLAIR abnormality. The priority is to prospectively identify biologically high-risk residual compartments and verify that the selected therapy reaches and modifies them. Local treatment should then be integrated with a distributed modality capable of controlling disease beyond the treated margin. In practical terms, the therapeutic target should shift from the visible tumor mass to the residual tumor–niche unit.

**Figure 5:**
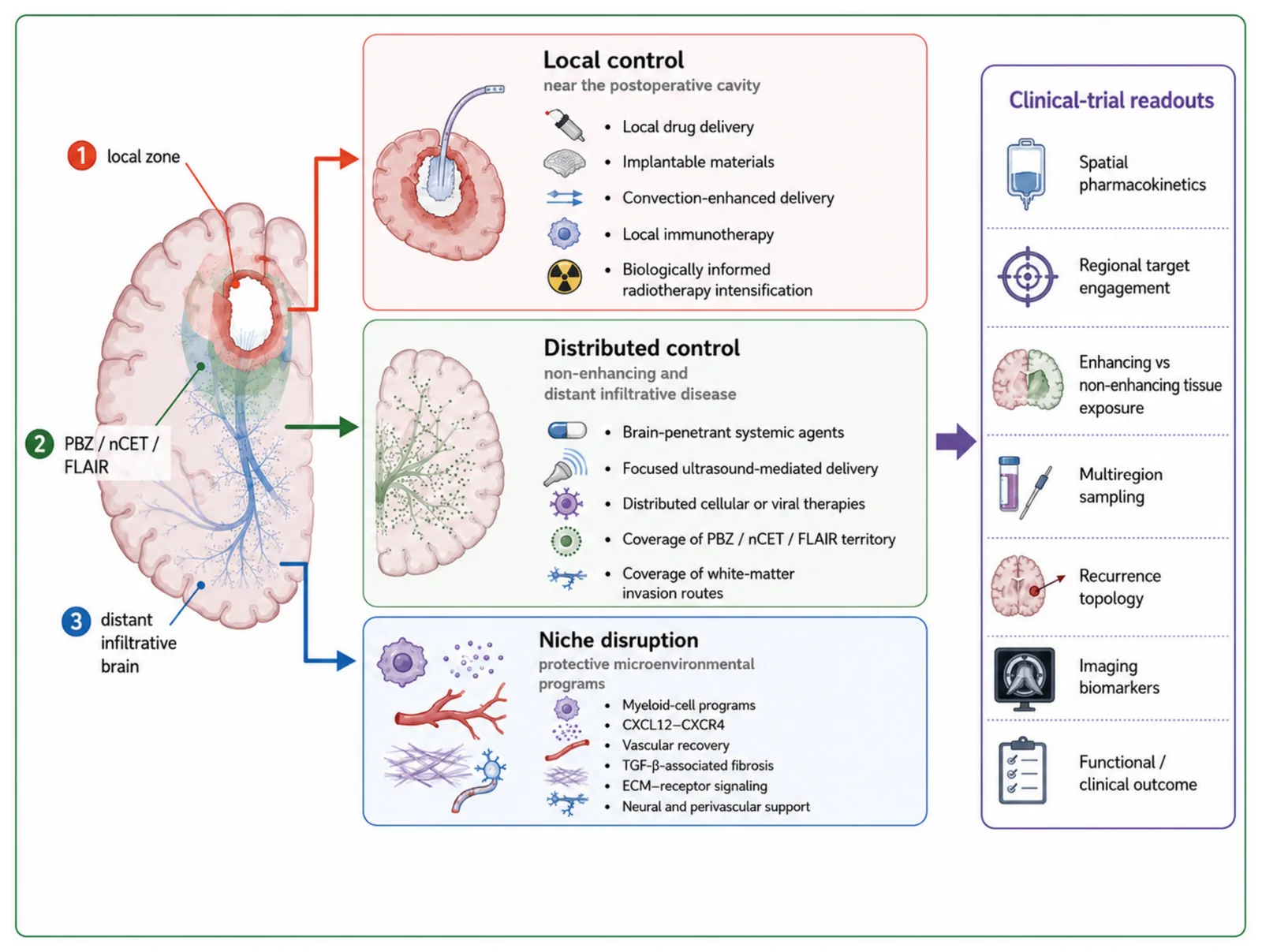
Spatially informed therapeutic and clinical-trial framework for residual glioblastoma. The schematic integrates local control near the postoperative cavity, distributed treatment of non-enhancing and distant infiltrative disease, and disruption of protective microenvironmental niches. Clinical evaluation should confirm spatial drug exposure and target engagement across enhancing and non-enhancing compartments using multiregion sampling, imaging biomarkers, and recurrence mapping. Drug delivery to the enhancing tumor alone is insufficient if the intended target is located within the PBZ or nCET. The PBZ, nCET, and FLAIR-abnormal territory are overlapping but non-equivalent compartments and are shown schematically rather than to scale. Abbreviations: ECM, extracellular matrix; FLAIR, fluid-attenuated inversion recovery; nCET, non-contrast-enhancing tumor; PBZ, peritumoral brain zone.

These considerations support a spatially informed framework that integrates local, distributed, and niche-directed treatment with regional assessment of drug delivery, target engagement, and recurrence patterns, as summarized in Figure 5.

### 9.6. Clinical Challenges in Targeting the Invasive Front

Targeting the invasive front creates a narrow therapeutic window because residual malignant cells are intermingled with functioning brain, including eloquent cortex and long-range white-matter tracts. The radiographic target is also uncertain: nCET, PBZ, and FLAIR abnormality overlap but are not equivalent, and edema, gliosis, or treatment effect can mimic infiltration. A single biopsy or imaging biomarker may therefore underrepresent a spatially heterogeneous field [13,26,55].

Drug delivery is an additional barrier. Non-enhancing brain may retain a more intact blood–brain barrier, and human window-of-opportunity and phase 0 studies show lower or heterogeneous exposure outside enhancing tumor [25,179]. Local therapies cannot address cells that have dispersed beyond the treated margin, whereas wider treatment increases the risk of injury to normal brain. Clinical translation therefore requires image-guided patient selection, regional pharmacokinetic and pharmacodynamic confirmation in non-enhancing tissue when safely obtainable, and endpoints that distinguish local from distant failure while accounting for treatment-related changes in contrast enhancement [15,28].

## 10. Limitations of the Present Review

This narrative synthesis has several limitations. The literature is heterogeneous in anatomical definitions, imaging criteria, sampling, tissue processing, model systems, and endpoints; many mechanistic claims derive from preclinical models or small, spatially restricted human cohorts. Because the search was non-systematic, selection and publication bias cannot be excluded, and no formal risk-of-bias or certainty assessment was performed. Associations identified by single-cell and spatial analyses do not by themselves establish causal or clinically actionable dependencies.

## 11. Future Directions

Four priorities are now actionable. First, standardize image-registered definitions and sampling of nCET, the PBZ, and the leading edge so spatial results can be compared across cohorts. Second, build paired diagnostic–recurrent datasets from clinically indicated repeat surgery and integrate them with serial imaging. Third, validate candidate neural, vascular, immune, and ECM dependencies in adult IDH-wild-type GBM models and human tissue, explicitly distinguishing evidence derived from DMG or xenograft systems. Fourth, use phase 0 and window-of-opportunity trials to require regional unbound drug exposure and target engagement in non-enhancing tissue before efficacy expansion [15,25]. Spatial recurrence pattern, neurological function, toxicity to normal brain, progression-free survival, and overall survival should be incorporated as complementary endpoints. These steps would move the field from descriptive mapping toward testable, clinically actionable PBZ strategies.

## 12. Conclusions

The central message of this review is that the GBM invasive margin is a residual-disease habitat rather than a diluted extension of the tumor core. Infiltrating malignant cells occupy spatially heterogeneous neural, vascular, immune, hypoxic, and ECM-supported niches, and therapy can further remodel those niches. This explains why CET control alone is insufficient. Translation should therefore focus on three linked tasks: identifying biologically high-risk non-enhancing compartments, delivering therapy to those compartments, and verifying regional target engagement. Local margin control will probably need to be combined with distributed treatment of infiltrating cells and selective disruption of supportive niches. At present, no therapy selected specifically by a spatial or molecular PBZ biomarker has shown an overall-survival benefit in a randomized trial; prospective validation remains the key next step.

## Figures and Tables

**Table 1:** Major cellular components of the glioblastoma invasive-margin ecosystem. Representative references are provided; additional supporting evidence is discussed in the corresponding sections of the main text.

Cellular Component	Principal Contribution	Representative Signals or Programs/Refs.
Infiltrating malignant cells	Low-density survival, invasion, cell-state transitions, and recurrence initiation	NPC-like, OPC-like, AC-like, MES-like, radial glia-like, cycling, and stress-adapted programs [16,100]
Reactive astrocytes	Survival, migration, stem-like maintenance, immune modulation, and treatment protection	Cx43; IL-6, TGF-β, TNF-α; tenascin-C; and osteopontin–CD44 signaling [65,67]
Microglia and monocyte-derived macrophages	Immunosuppression, matrix remodeling, vascular dysfunction, angiogenesis, and fibrosis	IL-10, TGF-β, MMP-9, VEGF, IDO, adrenomedullin, and spatial myeloid programs [57,101]
Endothelial and perivascular cells	Vessel co-option, angiocrine support, migration guidance, and stem-like cell protection	VEGF-A, Endocan–PDGFRα, CXCL12, perivascular ECM, and barrier remodeling [102,103]
Stromal-, fibroblast-like, and pericyte populations	Matrix deposition, fibrotic encapsulation, angiogenic support, and tissue mechanics	Collagens, TGF-β, tenascin-C, filamin-C, and endothelial-to-mesenchymal programs [104,105]
T lymphocytes	Immune surveillance limited by exhaustion, suppressive signaling, and hypoxic sequestration	PD-1/PD-L1, regulatory T cells, IL-10, TGF-β, and hypoxia-associated dysfunction [89,106]
Neurons and oligodendroglial-lineage cells	Neural substrates and signals supporting peri-axonal, white-matter, and neuron-rich invasion	Synaptic, axon-guidance, developmental, and disease-associated oligodendroglial programs [107,108]

Abbreviations: AC-like, astrocyte-like; CD44, cluster of differentiation 44; Cx43, connexin 43; CXCL12, C-X-C motif chemokine ligand 12; ECM, extracellular matrix; IDO, indoleamine 2,3-dioxygenase; IL, interleukin; MES-like, mesenchymal-like; MMP-9, matrix metalloproteinase-9; NPC-like, neural progenitor cell-like; OPC-like, oligodendrocyte progenitor cell-like; PD-1, programmed cell death protein 1; PD-L1, programmed death-ligand 1; PDGFRα, platelet-derived growth factor receptor alpha; TGF-β, transforming growth factor beta; TNF-α, tumor necrosis factor alpha; VEGF, vascular endothelial growth factor; VEGF-A, vascular endothelial growth factor A.

**Table 2:** Malignant cell states and programs relevant to glioblastoma invasion. Representative references are provided; additional supporting evidence is discussed in the corresponding sections of the main text.

State or Program	Key Molecular and Spatial Features	Interpretation and Limitations/Refs.
NPC-like	*CDK4* amplification; neural progenitor programs; diffuse or neuron-associated infiltrated regions	May support adaptation to low-density neural tissue; not a universal invasive state [100,121]
OPC-like	*PDGFRA* amplification; oligodendrocyte-progenitor programs; selected core/perivascular regions and distal peri-axonal invasion	Context-dependent role spanning core localization and long-range invasion [100,122]
AC-like	*EGFR* alterations; astrocytic programs; neuron-rich infiltrated brain and diffuse invasion	May facilitate tumor–neural interactions; spatial clustering has been associated with outcome [100,121]
MES-like	*NF1* loss; inflammatory, hypoxic, and stress-response programs; perinecrotic and local perivascular niches	Often linked to treatment adaptation, but not universally enriched at recurrence [21,100]
Radial glia-like/developmental	*HOPX*, *PTPRZ1*, *VIM*, and *TNC*; neuron-rich leading edge and infiltrated neural tissue	Supports developmental invasion; *FAM20C* evidence is mainly from DMG and requires adult-GBM validation [123,124]
Cycling and stress-adapted programs	Cell-cycle, AP-1, hypoxia, DNA-damage, and metabolic-stress modules; core or treatment-remodeled regions	Dynamic programs can coexist with lineage-like states and shift after therapy [22,125]

Abbreviations: AC-like, astrocyte-like; AP-1, activator protein 1; *CDK4*, cyclin-dependent kinase 4 gene; DMG, diffuse midline glioma; DNA, deoxyribonucleic acid; *EGFR*, epidermal growth factor receptor gene; *FAM20C*, Golgi-associated secretory pathway kinase gene; *HOPX*, HOP homeobox gene; MES-like, mesenchymal-like; *NF1*, neurofibromin 1 gene; NPC-like, neural progenitor cell-like; OPC-like, oligodendrocyte progenitor cell-like; *PDGFRA*, platelet-derived growth factor receptor alpha gene; *PTPRZ1*, protein tyrosine phosphatase receptor type Z1 gene; *TNC*, tenascin C gene; *VIM*, vimentin gene.

**Table 3:** Major anatomical routes of glioblastoma invasion. Representative references are provided; additional supporting evidence is discussed in the corresponding sections of the main text.

Route	Anatomical Substrate	Associated Programs or Regulators	Biological Implication/Refs.
Perivascular invasion and vessel co-option	Pre-existing or remodeled vessels and the perivascular space	MES-like and OPC-like states in selected models; ANXA1-associated vascular interaction; angiocrine signaling	Supports local spread and access to a therapy-protective vascular niche [102,121]
Peri-axonal and white-matter invasion	Axon-rich tracts, peri-axonal spaces, and the corpus callosum	OPC-like and distal-invasion-associated programs; neuronal and axon-guidance mechanisms	Enables long-range and contralateral dissemination beyond the immediate postoperative field [107,122]
Diffuse parenchymal/neuron-rich invasion	Relatively preserved gray matter containing neurons, astrocytes, oligodendroglia, microglia, and resident vessels	AC-like, NPC-like, and radial glia-like programs; *RFX4*, *HOPX*, and context-dependent effects of *FAM20C* depletion	Requires adaptation to low tumor-cell density and extensive tumor–neural communication [121,129]
Periventricular/subventricular attraction	Periventricular tissue and subventricular-zone-associated vascular niches	CXCL12–CXCR4-dependent migration of stem-like cells in experimental models	May provide a chemotactic and protective destination, but current evidence is predominantly preclinical [137,138]

Abbreviations: AC-like, astrocyte-like; ANXA1, annexin A1; CXCL12, C-X-C motif chemokine ligand 12; CXCR4, C-X-C motif chemokine receptor 4; *FAM20C*, Golgi-associated secretory pathway kinase gene; *HOPX*, HOP homeobox gene; MES-like, mesenchymal-like; NPC-like, neural progenitor cell-like; OPC-like, oligodendrocyte progenitor cell-like; *RFX4*, regulatory factor X4 gene.

**Table 4:** Spatial niches that support residual glioblastoma. Representative references are provided; additional supporting evidence is discussed in the corresponding sections of the main text.

Niche	Dominant Components	Key Mechanisms	Likely Consequence/Refs.
High-cerebral-blood-flow PBZ interface	Increased tumor-cell burden, neovascularization, macrophages, T cells, and altered endothelium	*VEGFA*, *EGFR*, *HIF1A*, *MMP9*, and PI3K–AKT/HIF-1 signaling	Candidate high-risk non-enhancing compartment [29,54]
Hypoxic/perinecrotic niche	Stress-adapted tumor cells, hypoxia-associated macrophages, sequestered cytotoxic T cells, and abnormal vessels	Mesenchymal/metabolic-stress programs, adrenomedullin-mediated vascular dysfunction, and immunosuppression	Resistant phenotypes, impaired drug delivery, and weak antitumor immunity [101,106]
Perivascular niche	Tumor, endothelial and mural cells; macrophages; fibroblast-like cells; astrocytic endfeet; and ECM	Angiocrine signaling, vessel co-option, osteopontin–CD44, Endocan–PDGFRα, and collagen remodeling	Stemness, radioprotection, migration, and recurrence-associated fibrosis [74,149]
Neuron-rich leading edge	Sparse tumor cells among neurons, astrocytes, oligodendroglia, microglia, and resident vessels	Developmental, synaptic, axon-guidance, and region-specific matrisome programs	Invasion through preserved brain and context-specific vulnerabilities [129,152]
Postoperative/fibrotic niche	Residual tumor cells, activated myeloid cells, senescent astrocytes, and vascular/fibroblast-like cells	Wound inflammation, senescence-associated secretory phenotype, TGF-β signaling, fibrosis, and immune exclusion	Dormancy, immune protection, and later regrowth [105,153]

Abbreviations: AKT, protein kinase B; CD44, cluster of differentiation 44; ECM, extracellular matrix; *EGFR*, epidermal growth factor receptor gene; HIF-1, hypoxia-inducible factor 1; *HIF1A*, hypoxia-inducible factor 1 alpha gene; *MMP9*, matrix metalloproteinase 9 gene; PBZ, peritumoral brain zone; PDGFRα, platelet-derived growth factor receptor alpha; PI3K, phosphatidylinositol 3-kinase; TGF-β, transforming growth factor beta; *VEGFA*, vascular endothelial growth factor A gene.

**Table 5:** Treatment-induced remodeling of the residual glioblastoma ecosystem. Representative references are provided; additional supporting evidence is discussed in the corresponding sections of the main text.

Intervention	Microenvironmental Response	Potential Protection of Residual Disease	Evidence/Refs.
Surgical resection	Transient blood–brain barrier disruption, wound inflammation, myeloid-cell recruitment, angiogenesis, and growth-factor release	Trophic support and proliferation of residual stem-like cells within an evolving postoperative niche	Human longitudinal observations and preclinical models [105,153,155]
Radiotherapy	Vascular injury, hypoxia, senescent astrocytes, senescence-associated secretory signaling, and recruitment of inflammatory myeloid cells	Rapid emergence of survival-promoting neighborhoods, revascularization, and tumor regrowth	Spatial preclinical studies and mechanistic models [105,154,156,157]
Temozolomide	Selection and reprogramming of malignant states, treatment-associated mutational damage, and changes in immune composition	Patient-specific recurrent trajectories; hypermutation in a subset of treated tumors	Human longitudinal cohorts and experimental models [21,22,172]
CSF-1R inhibition	Incomplete myeloid suppression followed by TGF-β- and neuroinflammation-dependent fibrotic remodeling	Encapsulation of surviving cells, dormancy, and restriction of immune surveillance	Limited clinical activity with direct preclinical evidence of compensatory fibrosis [105,159]

Abbreviations: CSF-1R, colony-stimulating factor 1 receptor; TGF-β, transforming growth factor beta.

**Table 6:** Selected therapeutic strategies for residual and invasive glioblastoma. Representative references are provided; additional supporting evidence is discussed in the corresponding sections of the main text.

Strategy	Primary Objective	Evidence Level	Key Limitation/Refs.
Biologically guided resection and radiotherapy	Reduce disease in safely accessible or imaging-defined high-risk regions	Clinical standard; spatial refinements under investigation	Neurological risk and limited specificity of FLAIR-based targeting [31,55]
Focused ultrasound and blood–brain barrier modulation	Improve drug delivery to non-enhancing or otherwise protected regions	Feasible in early-phase trials	Agent-specific benefit; uncertain regional pharmacokinetics and survival effect [189,190,191]
Local delivery platforms	Sustain high exposure at the recurrence-prone cavity margin	Clinical precedent; growing preclinical/early clinical evidence	Limited coverage of white-matter, perivascular, or distant infiltration [192,200]
CXCL12–CXCR4 blockade with or without VEGF inhibition	Limit post-radiation vascular recovery, invasion, and myeloid recruitment	Strong preclinical rationale; small phase I/II signals	Small non-randomized cohorts; no established survival benefit [166,168,169,170]
Locoregional cellular and oncolytic therapies	Deliver immune or viral therapy to the cavity, tumor, or cerebrospinal fluid	Phase I/II activity signals	Transient responses, antigen escape, limited persistence, and local immunosuppression [193,194,195]
Tumor-treating fields	Provide sustained postoperative treatment beyond the surgical cavity	Randomized overall-survival benefit with maintenance temozolomide	Not margin-specific; efficacy depends on adherence [196,197,198]
Multi-compartment or dual-target combinations	Target malignant cells and supportive immune, vascular, or stromal niches simultaneously	Promising preclinical and early clinical concepts	Toxicity, delivery, biomarker selection, and proof of simultaneous target engagement [168,194,199,200]

Abbreviations: CXCL12, C-X-C motif chemokine ligand 12; CXCR4, C-X-C motif chemokine receptor 4; FLAIR, fluid-attenuated inversion recovery; VEGF, vascular endothelial growth factor.

## Data Availability

No new data were created or analyzed in this study. Data sharing is not applicable to this article.

## Abbreviations

The following abbreviations are used in this manuscript:

5-ALA	5-aminolevulinic acid
AC-like	astrocyte-like
CAR-T	chimeric antigen receptor T-cell
CET	contrast-enhancing tumor
CNS	central nervous system
DMG	diffuse midline glioma
ECM	extracellular matrix
ESTRO–EANO	European Society for Radiotherapy and Oncology–European Association of Neuro-Oncology
FLAIR	fluid-attenuated inversion recovery
GBM	glioblastoma
GLASS	Glioma Longitudinal Analysis Consortium
L-RNA	L-ribonucleic acid
MDSC/MDSCs	myeloid-derived suppressor cell(s)
MES-like	mesenchymal-like
MRI	magnetic resonance imaging
mRANO	modified Response Assessment in Neuro-Oncology
nCET	non-contrast-enhancing tumor
NPC-like	neural progenitor cell-like
OPC-like	oligodendrocyte progenitor cell-like
PBZ	peritumoral brain zone
PET	positron emission tomography
RANO	Response Assessment in Neuro-Oncology
RGD	Arg-Gly-Asp
RNA	ribonucleic acid
scRNA-seq	single-cell RNA sequencing
snRNA-seq	single-nucleus RNA sequencing
TME	tumor microenvironment
Tregs	regulatory T cells
